# Supplementary material for: Cost-effectiveness of a domestic violence and abuse training and support programme in primary care in the real world: updated modelling based on an MRC phase IV observational pragmatic implementation study
Source: BMJ Open. 2018 Aug 29;8(8):e021256. doi: 10.1136/bmjopen-2017-021256 (PMC6119435; doi:10.1136/bmjopen-2017-021256)
Supplement: Author's manuscript [file bmjopen-2017-021256.draft_revisions.pdf]

BMJ Open is committed to open peer review. As part of this commitment we make the peer review history of every article we publish publicly available.

When an article is published we post the peer reviewers' comments and the authors' responses online. We also post the versions of the paper that were used during peer review. These are the versions that the peer review comments apply to.

The versions of the paper that follow are the versions that were submitted during the peer review process. They are not the versions of record or the final published versions. They should not be cited or distributed as the published version of this manuscript.

BMJ Open is an open access journal and the full, final, typeset and author-corrected version of record of the manuscript is available on our site with no access controls, subscription charges or pay-per-view fees (http://bmjopen.bmj.com).

If you have any questions on BMJ Open's open peer review process please email info.bmjopen@bmj.com

BMJ Open

# Cost-effectiveness of a domestic violence and abuse training and support programme in primary care in the real world: updated modelling based on a MRC phase IV observational pragmatic implementation study



| | |
|---|---|
| Journal: | *BMJ Open* |
| Manuscript ID | bmjopen-2017-021256 |
| Article Type: | Research |
| Date Submitted by the Author: | 21-Dec-2017 |





| | |
|---|---|
| Complete List of Authors: | Capelas Barbosa, Estela; University College London, Department of Applied Health Research<br>Verhoef, Talitha; University College London, Departmet of Applied Health Research<br>Morris, Stephen; University College London, Department of Applied Health Research<br>Solmi, Francesca; University College London, Division of Psychiatry; University College London<br>Johnson, Medina; IRISi<br>Sohal, Alex; Queen Mary University of London, Centre for Primary Care and Public Health<br>El-Shogri, Farah; Queen Mary University of London<br>Ronalds, Clare; Manchester Women's Aid, Pankhurst Trust Incorporating<br>Griffiths, Chris; Barts and The London School of Medicine and Dentistry,<br>Eldridge, Sandra; Queen Mary University of London, Centre for Primary Care and Public Health<br>Lewis, Natalia; Queen Mary University of London, Centre for Primary Care and Public Health<br>Devine, A; University of Oxford , Centre forTropical Medicine and Global Health; Mahidol University Phayathai Campus, Mahidol-Oxford Tropical Medicine Research Unit<br>Spencer, Anne; University of Exeter Medical School<br>Feder, Gene; University of Bristol, Community based medicine |





| | |
|---|---|
| Keywords: | Domestic violence, intimate partner violence, training programme, general practice, family medicine primary care, cost-effectiveness |



SCHOLARONE™
Manuscripts

**Title**

**Cost-effectiveness of a domestic violence and abuse training and support programme in primary care in the real world: updated modelling based on a MRC phase IV observational pragmatic implementation study**


Estela C. Barbosa (Corresponding author)
Department of Applied Health Research, UCL
Gower Street,London, UK WC1E 6BT
020 3108 3024
Email: e.barbosa@ucl.ac.uk

Talitha I. Verhoef
Department of Applied Health Research, UCL
1-19 Torrington Pl, London WC1E 7HB
United Kingdom

Steve Morris
Department of Applied Health Research, UCL
Gower Street,London, UK WC1E 6BT
020 3108 3024

Francesca Solmi
Division of Psychiatry, UCL
149 Tottenham Court Road, London W1T 7NF
United Kingdom

Medina Johnson
IRISi Interventions
PO Box 174 | Chepstow | NP16 9BT
United Kingdom

Alex Sohal
NIHR CLAHRC North Thames at Bart's Health NHS Trust, Centre for Primary Care and Public Health, Queen Mary University of London
58 Turner St, London,
United Kingdom EC1 5AT

Farah El-Shoghri
NIHR CLAHRC North Thames at Bart's Health NHS Trust, Centre for


For peer review only

Primary Care and Public Health, Queen Mary University of London
58 Turner St, London,
United Kingdom EC1 5AT

Clare Ronalds
IRIS GP Project Lead Manchester, Pankhurst Trust Incorporating Manchester Women's Aid
60-62 Nelson Street, Manchester M13 9WP
United Kingdom

Chris Griffiths
NIHR CLAHRC North Thames at Bart's Health NHS Trust, Centre for Primary Care and Public Health, Queen Mary University of London
58 Turner St, London,
United Kingdom EC1 5AT

Sandra Eldridge
NIHR CLAHRC North Thames at Bart's Health NHS Trust, Centre for Primary Care and Public Health, Queen Mary University of London
58 Turner St, London,
United Kingdom EC1 5AT

Natalia V. Lewis
NIHR CLAHRC North Thames at Bart's Health NHS Trust, Centre for Primary Care and Public Health, Queen Mary University of London
58 Turner St, London,
United Kingdom EC1 5AT

Angela Devine
Mahidol-Oxford Tropical Medicine Research Unit, Mahidol University and Centre forTropical Medicine and Global Health, Nuffield Department of Clinical Medicine, University of Oxford

Anne Spencer
University of Exeter Medical School
St Luke's Campus
Heavitree Road, Exeter EX1 2LU
United Kingdom

For peer review only

| | |
|---|---|
| Gene Feder | School of Social and Community Medicine, University of Bristol<br>39 Whatley Road, Bristol BS8 2PS<br>United Kingdom |

| | |
|---|---|
| Key words | Domestic violence, intimate partner violence, training programme, general practice, family medicine primary care, cost-effectiveness. |
| Word count | Abstract: 289<br>3418 (excluding title page, abstract, tables and acknowledgements and references)<br>Destination journal: BMJ Open, word limit: 3000-4000, structured abstract up to 300 words, up to 5 tables and figures. |

For peer review only

**Abstract**

*Objectives*:

To evaluate the cost-effectiveness of the implementation of the IRIS programme using up-to-date real-world information on costs and effectiveness from routine clinical practice. A Markov model was constructed to estimate mean costs and quality-adjusted life-years (QALYs) of IRIS versus usual care per woman registered at a general practice from a societal and health service perspective with a ten-year time horizon.

*Design and Setting*:

Cost–utility analysis in UK general practices, including data from six sites which have been running IRIS for at least two years across England.

*Participants*:

Based on the Markov model, we stipulated a hypothetical cohort of 10,000 women aged 16 years or older.

*Interventions*

The Identification and Referral to Improve Safety (IRIS) was a randomised controlled trial that tested the effectiveness of a primary care training and support intervention to improve the response to women experiencing DVA, and found it to be cost-effective. As a result, the IRIS programme has been implemented across the UK, generating data on costs and effectiveness outside a trial context.

*Results*:

The IRIS programme saved £14 per woman aged 16 or older registered in general practice (95% uncertainty interval [-£151; £37]) and produced QALY gains of 0.001 per woman (95% uncertainty interval [-0.005; 0.006]). The incremental net monetary benefit was positive both from a societal and NHS perspective (£42 and £22 respectively) and the IRIS programme was

For peer review only

cost-effective in 61% of simulations using real life data when the cost-effectiveness threshold was £20 000 per QALY gained as advised by NICE.

*Conclusion*:

The IRIS programme is likely to be cost-effective and cost-saving from a societal perspective in the UK and cost effective from a health service perspective, though there is considerable uncertainty surrounding these results, reflected in the large uncertainty intervals.

For peer review only

**Strengths and limitations of this study**

- We have used up-to-date routine data from several sites across England to evaluate the value for money of IRIS, a domestic violence training programme.
- We were unable to include any impact of the IRIS programme on children exposed to DVA, as to our knowledge, there are no available cohort studies focusing on the cost and benefits of DVA interventions for this population.
- Using up-to-date data on costs and effectiveness from routine clinical practice the national implementation of the IRIS programme is likely to be cost-effective and even cost-saving.

## Introduction

The lifetime prevalence of domestic violence and abuse (DVA) against women varies internationally from 15% to 71% (1). In the United Kingdom, in the year ending March 2017, 7.5% of women (1.2 million) experienced domestic abuse (2). Women who experience DVA suffer chronic health problems including gynaecological problems, gastrointestinal disorders, neurological symptoms, chronic pain, cardiovascular conditions and mental health problems (3-6). In 2012, the cost of DVA in the UK, including medical and social services, lost economic output and emotional costs, was estimated to be £11 billion (7). While such estimates highlight the importance of DVA as a public health and clinical problem, information on cost-effectiveness is needed to make an economic case for investment in DVA interventions in health care, particularly when health systems are dominated by austerity.

The Identification and Referral to Improve Safety (IRIS) trial tested the effectiveness of a training and support intervention for general practice teams in two English cities (8). Discussions about DVA between clinicians and patients were 22 times greater in the intervention practices compared with the control practices. Primary care practices that delivered the intervention also experienced a 6 fold and 3 fold increase in referrals received by DVA agencies and DVA-related notes in the patient medical records, respectively. The IRIS programme can now be commissioned across the UK: as of December 2016, 34 UK areas had commissioned IRIS; more than 800 GP practices nationally have had IRIS training, and over 5,000 women have been referred in to DVA support services by IRIS since 2010.

The cost-effectiveness of the IRIS trial was assessed using data from the trial and the programme was estimated to be good value for money (9). Given its national implementation, IRIS became a real-life, long-term intervention, raising the need for a new economic

For peer review only

evaluation outside the trial context. The aim of this study was to evaluate the cost-effectiveness of the IRIS programme now that it has been implemented across the UK. Our estimates use up-to-date figures from an MRC phase IV observational pragmatic implementation study (10) on costs and effectiveness from routine clinical practice and the most up-to-date model input parameters, including a recently updated Cochrane review of domestic violence advocacy (11).

## Methods

*Overview of economic evaluation*

This was a cost–utility analysis, comparing IRIS with usual care in general practices. The outcome measure was quality-adjusted life years (QALYs), as recommended for economic evaluations in the UK (12). The main analysis was from a societal perspective, as many of the costs of DVA are borne outside the health system; we also estimated cost utility from an NHS perspective. Costs were calculated in 2015/16 UK£. We calculated costs and benefits over a 10-year time horizon, with future costs and outcomes discounted at an annual rate of 3.5% (12).

*Model structure*

We developed a Markov model (Figure1) based on the previous analysis (9). The model has five states and the cycle length was six months; this length was chosen as it reflects the average amount of time women stay in contact with DVA advocacy services. A hypothetical cohort of 10,000 women aged 16 years or older was simulated moving between the states (Figure 1). Other than death, which is an absorbing state, women can transition between each of the other states 'Not abused', 'Abused but not identified', 'Abused and identified, seeing advocate educator', 'Abuse and identified, not seeing advocate educator'.


For peer review only - http://bmjopen.bmj.com/site/about/guidelines.xhtml

*Intervention*

The IRIS programme is a multi-component intervention that has been described in detail elsewhere (8, 9). In brief, it consists of two two-hour multidisciplinary training sessions, for the practice clinical team and one hour training for reception and ancillary staff. They are delivered jointly by an IRIS advocate educator from a local collaborating specialist DVA agency, alongside a clinician interested in DVA, the IRIS clinical lead. The advocate educator is central to the intervention, combining a training and support role to the practices with provision of advocacy to women referred. Other intervention components include a HARK template (13) in the electronic medical record triggered by entry of clinical problem codes (such as depression, anxiety, irritable bowel syndrome, pelvic pain and assault), an explicit referral pathway to a named IRIS advocate educator, and publicity materials about DVA visible in practices. Patients referred to the advocate educator are usually seen at the referring general practice, enhancing safety and confidentiality.

*Prevalence of domestic abuse*

The proportion of women aged 16 years or older experiencing abuse was estimated based on published epidemiological data. This was taken from a cross sectional study carried out by Richardson and colleagues in east London (14), which reported a prevalence of 0.17 or 17% in the population of women consulting a general practitioner or practice nurse. This is an estimate of the prevalence of DVA in general practice, generalizable for England. .

*Transition probabilities*

There are eight transitions between states in the model. Transition probabilities were obtained using observational data from the IRIS programme, the MOSAIC (MOthers' Advocates In the


For peer review only - http://bmjopen.bmj.com/site/about/guidelines.xhtml

Community) programme (8, 15), the Office for National Statistics (16, 17) and Health & Social Care Information Centre (18), and a Cochrane review (11), evaluating the reduction of any type of domestic abuse with any type of advocacy. Observational data were obtained from commissioned IRIS sites that have been running for two years or more, where there was at least one full-time equivalent advocate educator and 20 general practices trained. It included 6 clinical commissioning groups (CCGs) in northern England, south-west England and London. Given the inclusion criteria, the sites represent the implementation of the programme. . Table 1 provides the parameter values and their respective sources. Where no data were available, we have calculated estimates using the model calibration method described below.

*Model calibration*

Because of uncertainty surrounding transition probabilities from *Not abused* to *Abused but not identified* and *vice versa*, we used the prevalence of abuse (17%) estimated in Richardson and colleagues' study (14), to calibrate the model. The model was run for 3000 cycles, assuming that thereafter the number of women in each state would remain constant. This was based on our calculation of steady states. The transition probabilities from *Not abused* to *Abused but not identified* and *vice versa* were changed until the proportion of women in the *Not abused* state exactly reflected the observed prevalence (100-17=83%). The initial distribution of women in the three *Abused* states was also determined by this process.

*Utilities*

Each state in the Markov model was associated with a utility score, which consisted of a general measure of health-related quality-of-life (19), allowing us to measure QALYs associated with IRIS and the comparator based on the proportion of women in each health



For peer review only - http://bmjopen.bmj.com/site/about/guidelines.xhtml

state in each of the 20 6-monthly cycles in the model, totalling 10 years. The utility score of women who were not abused was assumed to be 0.85 (20). Wittenberg and colleagues conducted a cross-sectional survey to estimate community preferences for health states resulting from intimate partner violence. Using a UK-based algorithm, they found the utility of women experiencing any abuse was 0.64. When the severity/frequency of violence was low, the mean utility was 0.65 and when the severity/frequency was moderate or severe the mean utility was 0.63. For women who were abused in our model, we assumed this was moderate to severe, giving a utility score of 0.63 (21). For women seeing an advocate educator, we used the utility value of women with low abuse (0.65), implying that seeing an advocate educator slightly increased their quality-of-life scores.

*Costs*

We included: intervention costs, costs of onward referral, and costs associated with DVA (including costs to the UK National Health Service (NHS), lost economic output, costs to the criminal/civil justice system, and personal costs).

One IRIS advocate educator typically provides training, support and advocacy services for 24 general practices at any one point in time. Intervention costs were calculated based on the actual budget of the IRIS programme in the six sites (including advocate educator salaries, travel, recruitment, laptop, telephone, publicity, clinician consultancy, evaluation and central management costs) at a total six month cost across all sites of £272,613. This was divided by the number of registered women aged 16+ in IRIS-trained general practices in these sites (n=595,902). Costs of onward referral from the advocate educator was based on the finding of contact time from the IRIS trial, in which an onward referral was given to 57% of women in contact with an advocate educator and 63% of these women accepted this referral. Therefore,


For peer review only - http://bmjopen.bmj.com/site/about/guidelines.xhtml

although costs of onward referral were based on current budgets and salaries, the proportion of contact was obtained from the trial estimates. Total costs per onward referral were therefore £861. Taking into account the proportion of women given a referral and accepting it, and inflating it to 2015/16 UK£, average costs of advocate educator contact per abused woman were £312.

Costs associated with intimate partner violence in the UK are described by Walby and Olive (7). In their report, costs of lost economic output, health services, criminal justice system, civil justice system, social welfare, personal costs, specialised services and physical/emotional impact were individually reported, and total costs were €13,732 million (£11 billion) in 2012. We excluded costs of physical/emotional impact (€6,614 million), as they were not financial costs, but consisted of monetary valuing of health status, which in cost-effectiveness models ought to be captured in terms of QALYs; these were also not included in the original cost-effectiveness analysis. The remaining costs were converted to UK£ and inflated to 2015/16. Total costs per six months were £2,933 million. Based on the 2015 Crime Survey for England and Wales, it was estimated that 1.3 million women experienced intimate partner violence in 2015/16 in the UK (2). Mean costs per abused woman were therefore £2,043. We assumed that the costs of intimate partner abuse are similar to the costs of abuse by other family members, and that the costs would not differ between identified or unidentified abuse. In sensitivity analyses we have allowed the costs of identified abuse to increase or decrease by 10% compared to abuse that was not identified; similarly the costs of *Abused and identified, seeing advocate educator* were allowed to increase or decrease by 25%.

*Cost-utility analysis*

For peer review only - http://bmjopen.bmj.com/site/about/guidelines.xhtml

Costs and utilities were applied to each health state. Total costs and QALYs for the hypothetical cohort were generated for the IRIS programme and the control group. The main outcome was the incremental costs per QALY gained. In the UK an intervention is generally considered cost-effective when the incremental costs per QALY gained are less than £20,000 (12). We also presented the results of cost-effectiveness analysis in terms of incremental net monetary benefit (NMB). This was calculated as the mean incremental QALYs per woman registered at the general practice accruing to IRIS multiplied by the decision-makers' maximum willingness to pay for a QALY (assumed to be £20,000), minus the mean incremental cost per woman. Negative incremental NMBs indicate that usual care was preferred on cost-effectiveness grounds and positive incremental NMBs favour IRIS.

The cost-utility analysis was conducted using pooled national data, but we have also evaluated the cost-effectiveness at different local sites. We allowed all parameters, including costs and benefits, to vary across sites and reported them individually.

*Sensitivity analysis*

All parameters were varied in a one-way sensitivity analysis, using lower and upper limits based on 95% uncertainty intervals. We undertook a probabilistic sensitivity analysis, drawing random samples from the probability distributions of all parameters in 1,000 simulations. The proportion of simulations with an incremental cost per QALY gained below the cost-effectiveness threshold was calculated for different values, ranging from £0 to £50,000. The results were presented in a cost-effectiveness acceptability curve.

## Results

*Base case*



For peer review only - http://bmjopen.bmj.com/site/about/guidelines.xhtml

For peer review only

Parameter values used in the base case analysis are shown in Table 1. Over the ten-year time horizon, mean total costs per woman were £4,416 in the intervention group, compared to £4,430 in the control group (Table 2(a)). The IRIS programme therefore saves £14 per woman aged 16 and older registered to GP practices, from a societal perspective over 10 years. Total QALYs per woman were 0.001 higher in the intervention group (6.671) than in the control group (6.669). Because the intervention was associated with lower costs and greater effectiveness the incremental cost per QALY gained was negative (i.e. IRIS dominates current practice as it is both cost-saving and more effective than usual care) and the incremental NMB was positive (£42). The incremental NMB was also positive (£22) when using an NHS-only perspective (Table 2(b)).

Table 2 also presents the results for each site. The table shows that IRIS dominated current practice, from a societal perspective, in sites 1, 2, 3 and 4, with an incremental net monetary benefit (NMB) of £41, £89, £29 and £59 respectively. From a NHS perspective, only in site 1 did IRIS dominate current practice, although it was cost-effective, using the threshold advised by NICE of £20,000 per QALY gained, in sites 2 (ICER £2,585 per QALY gained), 3 (ICER £3,055 per QALY gained) and 4 (ICER £8,317 per QALY gained). IRIS was found to be cost-effective (ICER £5,882 per QALY gained) and borderline cost-effective (ICER £21,229 per QALY gained) from a societal and NHS perspectives respectively in site 5, and it was not cost-effective from either perspective in site 6 (ICER £52,557 per QALY gained and ICER £64,427 per QALY gained respectively).

*Sensitivity analyses*

Across all sites combined, results were most sensitive to varying the transition probability from *Abused but not identified* to *Not abused*. When in the control arm this was varied from

For peer review only

0.049 to 0.051, the incremental NMB varied from £110 to -£26 (Figure 2). When it was varied similarly in the intervention arm, the incremental NMB varied from -£25 to £109. Figure 2 shows the 12 parameters that when varied had the highest impact on the incremental NMB.

Incremental costs and QALYs varied widely in probabilistic sensitivity analyses. The 95% uncertainty interval for incremental costs was -£151 to £37, for incremental QALYs it was -0.005 to 0.006 and for the incremental NMB it was -£247 to £351. Figure 3(a) shows a scatter plot of the incremental costs and incremental QALYs from the 1,000 simulations. The IRIS programme is cheaper and more effective than the absence of the programme (usual care), dominating current practice in 35% of the simulations and was dominated by the absence of the programme in 18% of the simulations. The IRIS programme was cost-effective in 61% of simulations when the cost-effectiveness threshold was £20,000 (Figure 3(b)).

## Discussion

*Summary*

We found that the IRIS GP training and service programme is likely to be cost-effective and cost-saving in the UK compared to usual care. There is considerable uncertainty surrounding these results, but the probability that IRIS is cost-effective was more than 60% at the cost-effectiveness threshold commonly used in the UK. IRIS was more cost-effective when costs were measured from a societal perspective as the cost savings from reducing DVA were higher. IRIS was also cost-effective when taking an NHS-only perspective. There was some variation in value for money between sites.

*Comparison with existing literature*



For peer review only - http://bmjopen.bmj.com/site/about/guidelines.xhtml

We contacted researchers in the field and searched the NHS Economic Evaluations Database and the HTA Database at the Centre for Reviews and Dissemination (22) for cost-effectiveness analyses of DVA programmes using the search terms "domestic violence" and "cost*" (28/08/2017). We identified four economic impact studies, all using modelling methods: one based on the pilot of the IRIS trial (22), another based on the main trial (9), the third based on an evaluation of independent domestic violence Advisors (IDVA) (23), and the fourth of a trial of cognitive trauma therapy for abused women who have left the abusive relationship (23). All the studies found the interventions cost-effective, despite uncertainty. Our findings are consistent with these previous studies. Our study is the only one that analyses the economic impact of a primary care-based programme implemented outside of trial settings.

*Strengths and limitations*

Our analysis has the strength of being based on a previously published cost-effectiveness model, updated with real-life data. Importantly, intervention costs and the probability of referral with IRIS were based on actual clinical practice, rather than in a research setting. We also had new data for the probability of identifying abuse and for what happened to women who were abused in current practice without the programme. However, it was not possible to update all parameter values. In particular, we were unable to update the utility value estimates, although in the sensitivity analysis, we have allowed these to vary and results were relatively stable. Costs of the intervention were calculated by dividing the total costs of the programme over all registered women in practices with the IRIS programme. Many of these women will never experience abuse and therefore cannot directly benefit from the programme. If programme costs were divided over women experiencing abuse only, mean costs per woman would be higher. However, the QALYs gained would also be higher, as


For peer review only - http://bmjopen.bmj.com/site/about/guidelines.xhtml

these are also calculated for all women in the practices rather than just those who were abused. In fact we have attempted to calculate these results dividing cost and QALYs over women experiencing abuse and the final ICER was unchanged, as both the numerator and denominator change by the same proportion. We did not include any impact of the IRIS programme on children exposed to DVA, as to our knowledge, there are no available cohort studies focusing on the cost and benefits of DVA interventions for this population which might mean that we have underestimated the programme's cost-effectiveness. This was also highlighted in the NICE economic analysis of interventions to reduce incidence and harm of DVA: "It can be expected there are likely to be additional benefits such as [to] the children and wider family members of victims of domestic violence (p.11) (23).

Another limitation is that we have used mainly data on short-term outcomes, although modelled long-term outcomes. There is unfortunately little data on long-term outcomes of DVA and the effect of advocacy, although it is generally agreed that effects last for a long time.

*Implications for research and/or practice*

The IRIS programme is likely to be cost-effective and cost-saving when implemented in the real life of the in the UK National Health System. In order to decrease uncertainty around the cost-effectiveness estimates of IRIS and programmes like it, more data are needed on the utilities of women identified and women seeing an advocate and on long-term outcomes associated with DVA. Furthermore, future research should endeavour to understand the impacts and economic burden of DVA on exposed children, other family members and friends.

For peer review only

Finally, our study has shown that there is moderate variation in the value for money of IRIS across different sites, implying qualitative research could focus on identifying the causes of such variation, in order to reduce it.

For peer review only

**Authors' Contribution**

SM, CG, SE, AS and GF have designed the study. EB, TV, SM, FS and AD have developed the Markov model and carried out the analysis of data. AS, FES, CR, NL and MJ have collected and validated the data. EB and SM have produced the initial draft. All authors have critically revised the manuscript and approved the final version.

**Competing Interests disclosure**

MJ has been paid by the IRIS project since 2007 for employment as an IRIS Advocate Educator and then as a National Implementation Manager. She is currently paid by IRISi, a social enterprise that is promoting the commissioning of the IRIS programme, for employment as Chief Executive. GF reports grants from National Institute for Health Research (NIHR), during the conduct of the study; and he is a non-executive board member of IRISi. All other authors disclose no competing interests.

**Funding and Acknowledgements**

This research was funded by the National Institute for Health Research (NIHR) Collaboration for Leadership in Applied Health Research and Care North Thames at Barts Health NHS Trust.

GF acknowledges the support of the National Institute for Health Research from a programme grant for applied research RP-PG-0614-20012 REPROVIDE (Reaching Everyone Programme of Research on Violence in diverse Domestic Environments).

The views expressed are those of the author(s) and not necessarily those of the NHS, the NIHR or the Department of Health.



For peer review only - http://bmjopen.bmj.com/site/about/guidelines.xhtml

We would like to thank our IRIS partners who deliver the programme in the sites, especially those in northern England, south-west England and London who took the time and effort to provide us with data.

**Data sharing**

The anonymised data used in this study can be obtained from the corresponding author.

For peer review only

## References

1. Garcia-Moreno C, Jansen HA, Ellsberg M, Heise L, Watts CH. Prevalence of intimate partner violence: findings from the WHO multi-country study on women's health and domestic violence. Lancet. 2006;368(9543):1260-9.

2. ONS. Intimate personal violence and partner abuse compendium. 2016.

3. Bonomi AE, Anderson ML, Reid RJ, Rivara FP, Carrell D, Thompson RS. Medical and psychosocial diagnoses in women with a history of intimate partner violence. Archives of internal medicine. 2009;169(18):1692-7.

4. Campbell JC. Health consequences of intimate partner violence. Lancet. 2002;359(9314):1331-6.

5. Tollestrup K, Sklar D, Frost FJ, Olson L, Weybright J, Sandvig J, et al. Health indicators and intimate partner violence among women who are members of a managed care organization. Prev Med. 1999;29(5):431-40.

6. Coid J, Petruckevitch A, Chung WS, Richardson J, Moorey S, Feder G. Abusive experiences and psychiatric morbidity in women primary care attenders. The British journal of psychiatry : the journal of mental science. 2003;183:332-9; discussion 40-1.

7. Walby S, Olive P. Estimating the costs of gender-based violence in the European Union. European Institute for Gender Equality, 2014.

8. Feder G, Davies RA, Baird K, Dunne D, Eldridge S, Griffiths C, et al. Identification and Referral to Improve Safety (IRIS) of women experiencing domestic violence with a primary care training and support programme: a cluster randomised controlled trial. Lancet. 2011;378(9805):1788-95.

9. Devine A, Spencer A, Eldridge S, Norman R, Feder G. Cost-effectiveness of Identification and Referral to Improve Safety (IRIS), a domestic violence training and support programme for primary care: a modelling study based on a randomised controlled trial. BMJ open. 2012;2(3).

10. Sohal A, Dowrick A, El-Shoghri F, Beresford L, Lewis N, Barbosa E, et al. Improving the healthcare response to domestic violence and abuse in primary care: protocol for evaluation of a complex intervention's implementation into multiple general practices, including a phase IV observational segmented regression interrupted time series analysis BMJ Public Health. 2018 forthcoming.

11. Rivas C, Ramsay J, Sadowski L, Davidson LL, Dunne D, Eldridge S, et al. Advocacy interventions to reduce or eliminate violence and promote the physical and psychosocial well-

For peer review only

being of women who experience intimate partner abuse. status and date: New search for studies and content updated (no change to conclusions), published in. 2015(12).

12. NICE NIoHaCE. Guide to the methods of technology appraisal 2013. Available from: http://www.nice.org.uk/media/D45/1E/GuideToMethodsTechnologyAppraisal2013.pdf.

13. Sohal H, Eldridge S, Feder G. The sensitivity and specificity of four questions (HARK) to identify intimate partner violence: a diagnostic accuracy study in general practice. BMC family practice. 2007;8(1):49.

14. Richardson J, Coid J, Petruckevitch A, Chung WS, Moorey S, Feder G. Identifying domestic violence: cross sectional study in primary care. Bmj. 2002;324(7332):274.

15. Taft AJ, Small R, Hegarty KL, Watson LF, Gold L, Lumley JA. Mothers' AdvocateS In the Community (MOSAIC)--non-professional mentor support to reduce intimate partner violence and depression in mothers: a cluster randomised trial in primary care. BMC public health. 2011;11:178.

16. Statistics OoN. Crime Statistics, Focus on Violent Crime and Sexual Offences 2013/14 [4 November 2015]. Available from: http://www.ons.gov.uk/ons/rel/crime-stats/crime-statistics/focus-on-violent-crime-and-sexual-offences--2013-14/index.html.

17. Statistics OfN. Mortality Statistics: Deaths Registered in England and Wales 2013. Available from: http://www.ons.gov.uk/ons/publications/re-reference-tables.html?edition=tcm%3A77-327590.

18. Centre HSCI. Numbers of Patients Registered at a GP practice January 2014

19. Horsman J, Furlong W, Feeny D, Torrance G. The Health Utilities Index (HUI®): concepts, measurement properties and applications. Health and quality of life outcomes. 2003;1(1):54.

20. Kind K, Hardman G, Macran S. UK Population norms for EQ-5D. University of York, 1999.

21. Wittenberg E, Lichter EL, Ganz ML, McCloskey LA. Community preferences for health states associated with intimate partner violence. Medical care. 2006;44(8):738-44.

22. York Uo. Centre for Reviews and Dissemination. Available from: http://www.crd.york.ac.uk/CRDWeb/HomePage.asp.

23. Mallender J, Venkatachalam M, Onwude O, Jhita T. Economic analysis of interventions to reduce incidence and harm of domestic violence. London: National Institute for Health and Care Excellence. 2013.

For peer review only

**Table 1. Model input parameters: probabilities; utilities; and, costs.**

| Parameter | Base case value | Lower limit | Upper limit | Distribution | Source | IRIS trial base value[1] |
|---|---|---|---|---|---|---|
| ***Probabilities*** | | | | | | |
| Proportion of women experiencing abuse | 0.17 | 0.147 | 0.194 | Beta | (14) | 0.17 |
| *Starting distribution for women who are abused* | | | | | | |
| Abused and identified, seeing advocate educator | 0.003¶ | 0 | 0.0066 | Uniform | * | - |
| Abused and identified, not seeing advocate educator | 0.033¶ | 0 | 0.0660 | Uniform | * | - |
| Abused but not identified | 0.964¶ | - | - | Uniform | Complement | - |
| *Transition probabilities* | | | | | | |
| Not abused to Abused but not identified | 0.0037¶ | 0.0004 | 0.0106 | Dirichlet | * | 0.0075 |
| Not abused to Dead | 0.00551¶ | 0.0010 | 0.0136 | Dirichlet | (13, 15) | 0.0058 |
| Stay in Not abused | 0.9908¶ | - | - | Dirichlet | Complement | 0.9867 |
| Abused but not identified to Not abused (control) | 0.0500¶ | 0.0450 | 0.0553 | Dirichlet | * | 0.025 |
| Abused but not identified to Abused and identified, not seeing advocate educator (control) | 0.0027¶ | 0.0016 | 0.0040 | Dirichlet | IRIS-programme local sites | 0.0094 |



For peer review only - http://bmjopen.bmj.com/site/about/guidelines.xhtml

| | | | | | | |
|---|---|---|---|---|---|---|
| Abused but not identified to Abused and identified, seeing advocate educator (control) | 0.0005¶ | 0.0001 | 0.0011 | Dirichlet | IRIS-programme local sites | 0.0016 |
| Abused but not identified to Dead (control) | 0.00554¶ | 0.0039 | 0.0074 | Dirichlet | (13, 15) | 0.0059 |
| Stay in Abused but not identified (control) | 0.9444¶ | - | - | Dirichlet | Complement | 0.9581 |
| Abused but not identified to Not abused (intervention) | 0.0500¶ | 0.0450 | 0.0553 | Dirichlet | * | 0.025 |
| Abused but not identified to Abused and identified, not seeing advocate educator (intervention) | 0.0109¶ | 0.0086 | 0.0135 | Dirichlet | IRIS-programme local sites | 0.0207 |
| Abused but not identified to Abused and identified, seeing advocate educator (intervention) | 0.0056¶ | 0.0040 | 0.0076 | Dirichlet | IRIS-programme local sites | 0.0101 |
| Abused but not identified to Dead (intervention) | 0.00554¶ | 0.0039 | 0.0074 | Dirichlet | (6) | 0.0059 |
| Stay in Abused but not identified (intervention) | 0.9419 ¶ | - | - | Dirichlet | Complement | 0.9383 |
| Abused and identified, seeing advocate educator to Not abused | 0.1408¶ | 0.0707 | 0.2301 | Dirichlet | (15) | 0.0888 |
| Abused and identified, seeing advocate educator to Dead | 0.00554¶ | 0.0000 | 0.0309 | Dirichlet | (13, 15) | 0.0059 |
| Stay in Abused and identified, seeing advocate educator | 0.8536¶ | - | - | Dirichlet | Complement | 0.9053 |



For peer review only - http://bmjopen.bmj.com/site/about/guidelines.xhtml

| | | | | | | |
|---|---|---|---|---|---|---|
| Abused and identified, not seeing advocate educator to Not abused | 0.0781¶ | 0.0136 | 0.1912 | Dirichlet | (15) | 0.0717 |
| Abused and identified, not seeing advocate educator to Dead | 0.00554¶ | 0.0000 | 0.0438 | Dirichlet | (13, 15) | 0.0059 |
| Stay in Abused and identified, not seeing advocate educator | 0.9163¶ | - | - | Dirichlet | Complement | 0.9223 |
| ***Utilities*** | | | | | | |
| Not abused | 0.85 | 0.840 | 0.860 | Beta | (20) | - |
| Abused but not identified | 0.63 | 0.503 | 0.749 | Beta | (21) | - |
| Abused and identified, seeing advocate educator | 0.65 | 0.518 | 0.771 | Beta | (21) | - |
| Abused and identified, not seeing advocate educator | 0.63 | 0.503 | 0.749 | Beta | (21) | - |
| ***Costs*** | | | | | | |
| Costs of the intervention, per women registered, per 6 months | £0.46¶ | £0.01 | £1.69 | Gamma | IRIS-programme local sites | £0.55 |
| Cost of onward referral, once | £312¶ | £8 | £1127 | Gamma | IRIS-programme | £298 |



For peer review only - http://bmjopen.bmj.com/site/about/guidelines.xhtml

| | | | | | | |
|---|---|---|---|---|---|---|
| | | | | | local sites & (9) | |
| Cost of Abused but not identified | £2043 | £52 | £7536 | Gamma | (7) | £4721 |
| Weighted costs Abused and identified, seeing advocate educator | 1 | 0.75 | 1.25 | Gamma | Assumption | - |
| Weighted costs Abused and identified, not seeing advocate educator | 1 | 0.9 | 1.1 | Gamma | Assumption | - |

Costs are in 2015/16 UK£.
* Internal calculation based on model calibration.
¶ Value updated from Devine et al (9).
[1] Values obtained from Devine et al (9).



For peer review only - http://bmjopen.bmj.com/site/about/guidelines.xhtml

**Table 2. Base case results.**

| | (a) Societal perspective | | | (b) NHS-only perspective | | |
|---|---|---|---|---|---|---|
| **National IRIS (pooled results)** | **Costs** | **QALYs** | **Cost-effectiveness** | **Costs** | **QALYs** | **Cost-effectiveness** |
| Intervention (IRIS programme) | £4416 | 6.671 | | £1238 | 6. 671 | |
| Control (no programme) | £4430 | 6.669 | | £1232 | 6. 669 | |
| Difference (intervention vs. control) | -£14 | 0.001 | -ve (intervention dominates control) | £6 | 0.001 | £3913 per QALY gained |
| Incremental NMB* | | | £42 | | | £22 |
| **Local site 1** | | | | | | |
| Intervention (IRIS programme) | £4318 | 6.671 | | £1231 | 6.671 | |
| Control (no programme) | £4334 | 6.669 | | £1232 | 6.669 | |
| Difference (intervention vs. control) | -£16 | 0.001 | -ve (intervention dominates control) | -£1 | 0.001 | -ve (intervention dominates control) |
| Incremental NMB* | | | £41 | | | £26 |
| **Local site 2** | | | | | | |
| Intervention (IRIS programme) | £4305 | 6.673 | | £1240 | 6.673 | |
| Control (no programme) | £4333 | 6.670 | | £1232 | 6.670 | |
| Difference (intervention vs. control) | -£28 | 0.003 | -ve (intervention | £8 | 0.003 | £2585 per QALY |



For peer review only - http://bmjopen.bmj.com/site/about/guidelines.xhtml

| | | | | | | |
|---|---|---|---|---|---|---|
| | | | dominates control) | | | gained |
| Incremental NMB* | | | £89 | | | £54 |
| **Local site 3** | | | | | | |
| Intervention (IRIS programme) | £4325 | 6.671 | | £1235 | 6.671 | |
| Control (no programme) | £4334 | 6.670 | | £1232 | 6.670 | |
| Difference (intervention vs. control) | -£9 | 0.001 | -ve (intervention dominates control) | £3 | 0.001 | £3055 per QALY gained |
| Incremental NMB* | | | £29 | | | £17 |
| **Local site 4** | | | | | | |
| Intervention (IRIS programme) | £4326 | 6.672 | | £1253 | 6.672 | |
| Control (no programme) | £4334 | 6.669 | | £1232 | 6.669 | |
| Difference (intervention vs. control) | -£8 | 0.003 | -ve (intervention dominates control) | £21 | 0.003 | £8317 per QALY gained |
| Incremental NMB* | | | £59 | | | £30 |
| **Local site 5** | | | | | | |
| Intervention (IRIS programme) | £4337 | 6.670 | | £1244 | 6.670 | |
| Control (no programme) | £4332 | 6.669 | | £1232 | 6.669 | |
| Difference (intervention vs. control) | £4 | 0.001 | £5882 per QALY | £12 | 0.001 | £21229 per QALY |



For peer review only - http://bmjopen.bmj.com/site/about/guidelines.xhtml

For peer review only

| | | | | | | |
|---|---|---|---|---|---|---|
| | | | gained | | | gained |
| Incremental NMB* | | | £6 | | | £0 |
| **Local site 6** | | | | | | |
| Intervention (IRIS programme) | £4395 | 6.671 | | £1307 | 6.671 | |
| Control (no programme) | £4334 | 6.670 | | £1232 | 6.670 | |
| Difference (intervention vs. control) | £61 | 0.001 | £52557 per QALY gained | £75 | 0.001 | £64427 per QALY gained |
| Incremental NMB* | | | -£38 | | | -£52 |

NMB = net monetary benefit. QALY = quality-adjusted life year. Costs are in 2015/16 UK£. Numbers may not sum due to rounding.

*Measured at a willingness to pay for a QALY of £20 000.



For peer review only - http://bmjopen.bmj.com/site/about/guidelines.xhtml

**Figure 1. Health states and movement between health states in Markov model.**

Legend: The model starts with all women in either the ‘Not abused’ state or one of the states associated with abuse, based on the prevalence of DVA (see text). Women in the ‘Not abused’ state could stay in this state, move to ‘Abused but not identified’ or die from any cause. Once women were in the ‘Abused but not unidentified’ state, they could stay in that state, move back to ‘Not abused’, move to ‘Abused and identified, seeing advocate’ or ‘Abused and identified, not seeing advocate’ or die. Women in the ‘Abused and identified’ states could stay in these states, move back to ‘Not abused’ or die.

For peer review only



For peer review only - http://bmjopen.bmj.com/site/about/guidelines.xhtml

**Figure 2. Univariate sensitivity analysis.**

Legend: All analyses are as for the base case analysis with univariate adjustment of the parameters listed (see text). Results are point estimates of the incremental net monetary benefit (NMB) of the intervention vs. control. The incremental net monetary benefit is calculated at a maximum willingness to pay for a QALY of £20 000.

For peer review only



For peer review only - http://bmjopen.bmj.com/site/about/guidelines.xhtml

**Figure 3. Probabilistic sensitivity analysis.**

(a) **Scatter plot of incremental costs and incremental QALYs from 1000 simulations**
(b) **Cost-effectiveness acceptability curve showing the probability that the intervention is cost-effective vs. control at different values of the maximum willingness to pay for a QALY**

Legend: QALY = quality-adjusted life year. Costs are in 2015/16 UK£.

For peer review only



For peer review only - http://bmjopen.bmj.com/site/about/guidelines.xhtml

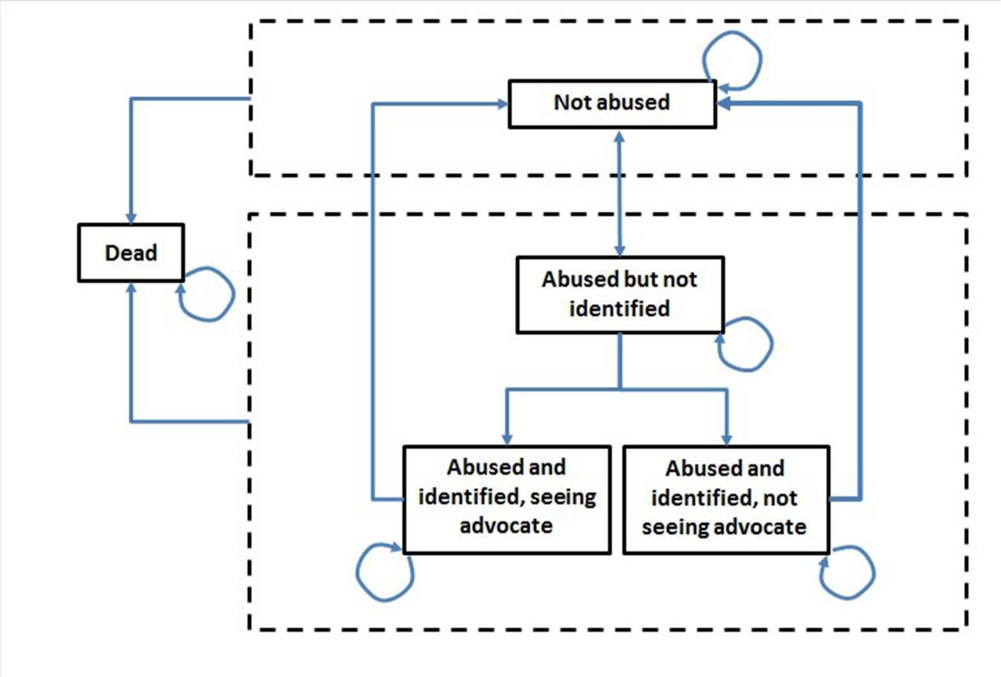


Figure 1. Health states and movement between health states in Markov model.
Legend: The model starts with all women in either the 'Not abused' state or one of the states associated with abuse, based on the prevalence of DVA (see text). Women in the 'Not abused' state could stay in this state, move to 'Abused but not identified' or die from any cause. Once women were in the 'Abused but not unidentified' state, they could stay in that state, move back to 'Not abused', move to 'Abused and identified, seeing advocate' or 'Abused and identified, not seeing advocate' or die. Women in the 'Abused and identified' states could stay in these states, move back to 'Not abused' or die.

203x137mm (96 x 96 DPI)

only

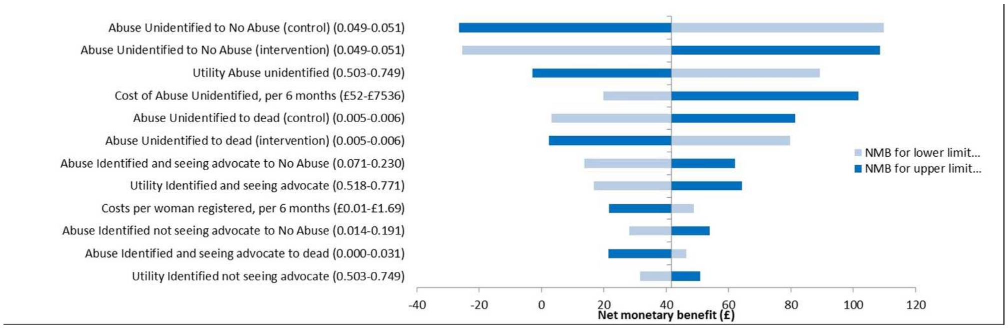


Figure 2. Univariate sensitivity analysis.
Legend: All analyses are as for the base case analysis with univariate adjustment of the parameters listed (see text). Results are point estimates of the incremental net monetary benefit (NMB) of the intervention vs. control. The incremental net monetary benefit is calculated at a maximum willingness to pay for a QALY of £20 000.

267x86mm (150 x 150 DPI)

For peer review only

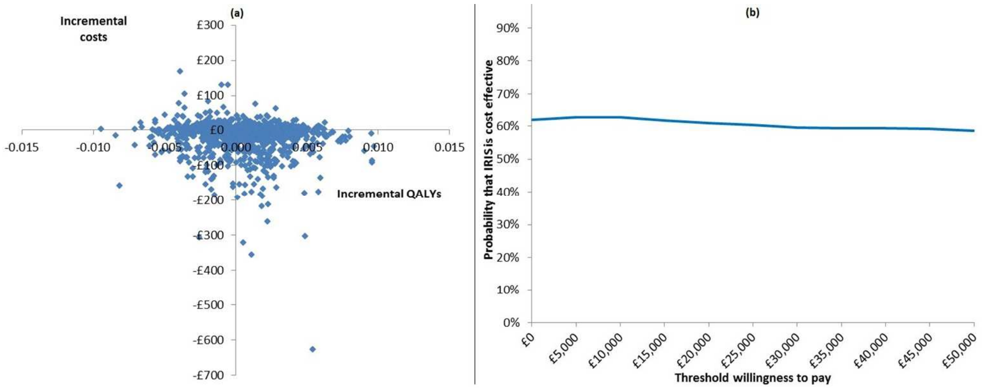


Figure 3. Probabilistic sensitivity analysis.
(a) Scatter plot of incremental costs and incremental QALYs from 1000 simulations
(b) Cost-effectiveness acceptability curve showing the probability that the intervention is cost-effective vs. control at different values of the maximum willingness to pay for a QALY
Legend: QALY = quality-adjusted life year. Costs are in 2015/16 UK£.

415x159mm (96 x 96 DPI)

For peer review only

# Cost-effectiveness of a domestic violence and abuse training and support programme in primary care in the real world: updated modelling based on a MRC phase IV observational pragmatic implementation study

| | |
|---|---|
| Journal: | *BMJ Open* |
| Manuscript ID | bmjopen-2017-021256.R1 |
| Article Type: | Research |
| Date Submitted by the Author: | 15-May-2018 |
| Complete List of Authors: | Capelas Barbosa, Estela; University College London, Department of Applied Health Research<br>Verhoef, Talitha; University College London, Departmet of Applied Health Research<br>Morris, Stephen; University College London, Department of Applied Health Research<br>Solmi, Francesca; University College London, Division of Psychiatry; University College London<br>Johnson, Medina; IRISi<br>Sohal, Alex; Queen Mary University of London, Centre for Primary Care and Public Health<br>El-Shogri, Farah; Queen Mary University of London<br>Dowrick, Susanna; Queen Mary University of London, Centre for Primary Care and Public Health<br>Ronalds, Clare; Manchester Women's Aid, Pankhurst Trust Incorporating<br>Griffiths, Chris; Barts and The London School of Medicine and Dentistry,<br>Eldridge, Sandra; Queen Mary University of London, Centre for Primary Care and Public Health<br>Lewis, Natalia; Queen Mary University of London, Centre for Primary Care and Public Health<br>Devine, A; University of Oxford , Centre forTropical Medicine and Global Health; Mahidol University Phayathai Campus, Mahidol-Oxford Tropical Medicine Research Unit<br>Spencer, Anne; University of Exeter Medical School<br>Feder, Gene; University of Bristol, Community based medicine |
| <b>Primary Subject Heading</b>: | Health economics |
| Secondary Subject Heading: | General practice / Family practice |
| Keywords: | Domestic violence, intimate partner violence, training programme, general practice, family medicine primary care, cost-effectiveness |
| | |

For peer review only

**Title** **Cost-effectiveness of a domestic violence and abuse training and support programme in primary care in the real world: updated modelling based on a MRC phase IV observational pragmatic implementation study**

Estela C. Barbosa (Corresponding author)
Department of Applied Health Research, UCL
Gower Street, London, UK WC1E 6BT
020 3108 3024
Email: e.barbosa@ucl.ac.uk

Talitha I. Verhoef
Department of Applied Health Research, UCL
1-19 Torrington Pl, London WC1E 7HB
United Kingdom

Steve Morris
Department of Applied Health Research, UCL
Gower Street, London, UK WC1E 6BT
020 3108 3024

Francesca Solmi
Division of Psychiatry, UCL
149 Tottenham Court Road, London W1T 7NF
United Kingdom

Medina Johnson
IRISi Interventions
PO Box 174 | Chepstow | NP16 9BT
United Kingdom

For peer review only

Alex Sohal — NIHR CLAHRC North Thames at Bart's Health NHS Trust, Centre for Primary Care and Public Health, Queen Mary University of London

58 Turner St, London,

United Kingdom EC1 5AT

Farah El-Shoghri — NIHR CLAHRC North Thames at Bart's Health NHS Trust, Centre for Primary Care and Public Health, Queen Mary University of London

58 Turner St, London,

United Kingdom EC1 5AT

Susanna Dowrick — NIHR CLAHRC North Thames at Bart's Health NHS Trust, Centre for Primary Care and Public Health, Queen Mary University of London

58 Turner St, London,

United Kingdom EC1 5AT

Clare Ronalds — IRIS GP Project Lead Manchester, Pankhurst Trust Incorporating Manchester Women's Aid

60-62 Nelson Street, Manchester M13 9WP

United Kingdom

Chris Griffiths — NIHR CLAHRC North Thames at Bart's Health NHS Trust, Centre for Primary Care and Public Health, Queen Mary University of London

58 Turner St, London,

United Kingdom EC1 5AT


For peer review only

Sandra Eldridge
NIHR CLAHRC North Thames at Bart's Health NHS Trust, Centre for Primary Care and Public Health, Queen Mary University of London

58 Turner St, London,

United Kingdom EC1 5AT

Natalia V. Lewis
NIHR CLAHRC North Thames at Bart's Health NHS Trust, Centre for Primary Care and Public Health, Queen Mary University of London

58 Turner St, London,

United Kingdom EC1 5AT

Angela Devine
Mahidol-Oxford Tropical Medicine Research Unit, Mahidol University and Centre for Tropical Medicine and Global Health, Nuffield Department of Clinical Medicine, University of Oxford

Anne Spencer
University of Exeter Medical School

St Luke's Campus

Heavitree Road, Exeter EX1 2LU

United Kingdom

Gene Feder
School of Social and Community Medicine, University of Bristol

39 Whatley Road, Bristol BS8 2PS

United Kingdom

For peer review only

| | |
|---|---|
| Key words | Domestic violence, intimate partner violence, training programme, general practice, family medicine primary care, cost-effectiveness. |
| Word count | Abstract: 300 |
| | 4006 (excluding title page, abstract, tables and acknowledgements and references) |

For peer review only

**Abstract**

*Objectives*:

To evaluate the cost-effectiveness of the implementation of the Identification and Referral to Improve Safety (IRIS) programme using up-to-date real-world information on costs and effectiveness from routine clinical practice. A Markov model was constructed to estimate mean costs and quality-adjusted life-years (QALYs) of IRIS versus usual care per woman registered at a general practice from a societal and health service perspective with a ten-year time horizon.

*Design and Setting*:

Cost–utility analysis in UK general practices, including data from six sites which have been running IRIS for at least two years across England.

*Participants*:

Based on the Markov model, which uses health states to represent possible outcomes of the intervention, we stipulated a hypothetical cohort of 10,000 women aged 16 years or older.

*Interventions*

The IRIS trial was a randomised controlled trial that tested the effectiveness of a primary care training and support intervention to improve the response to women experiencing DVA, and found it to be cost-effective. As a result, the IRIS programme has been implemented across the UK, generating data on costs and effectiveness outside a trial context.

*Results*:

The IRIS programme saved £14 per woman aged 16 or older registered in general practice (95% uncertainty interval [-£151; £37]) and produced QALY gains of 0.001 per woman (95% uncertainty interval [-0.005; 0.006]). The incremental net monetary benefit was positive both from a societal and NHS perspective (£42 and £22 respectively) and the IRIS programme was

For peer review only

cost-effective in 61% of simulations using real life data when the cost-effectiveness threshold was £20 000 per QALY gained as advised by NICE.

*Conclusion*:

The IRIS programme is likely to be cost-effective and cost-saving from a societal perspective in the UK and cost effective from a health service perspective, though there is considerable uncertainty surrounding these results, reflected in the large uncertainty intervals.

**Strengths and limitations of this study**

- We have used up-to-date routine data from several sites across England to evaluate the value for money of IRIS, a domestic violence training programme.
- We were unable to include any impact of the IRIS programme on children exposed to DVA, as to our knowledge, there are no available cohort studies focusing on the cost and benefits of DVA interventions for this population.
- We have used mainly data on short-term outcomes, although modelled long-term outcomes, as to our knowledge, no study has tracked women subject to DVA over long periods of time.

For peer review only

## Introduction

The lifetime prevalence of domestic violence and abuse (DVA) against women, as defined by the United Nations (1), varies internationally from 15% to 71% (2). In the United Kingdom, in the year ending March 2017, 7.5% of women (1.2 million) experienced domestic abuse (3). Women who experience DVA suffer chronic health problems including gynaecological problems, gastrointestinal disorders, neurological symptoms, chronic pain, cardiovascular conditions and mental health problems (4-7). In 2012, the cost of DVA in the UK, including medical and social services, lost economic output and emotional costs, was estimated to be £11 billion (8). While such estimates highlight the importance of DVA as a public health and clinical problem, information on cost-effectiveness is needed to make an economic case for investment in DVA interventions in health care, particularly when health systems are dominated by austerity.

The Identification and Referral to Improve Safety (9) trial tested the effectiveness of a training and support intervention for general practice teams in two English cities (10). Discussions about DVA between clinicians and patients were 22 times greater in the intervention practices compared with the control practices. Primary care practices that delivered the intervention also experienced a 6 fold and 3 fold increase in referrals received by DVA agencies and DVA-related notes in the patient medical records, respectively. The IRIS programme can now be commissioned across the UK: as of December 2016, 34 UK areas had commissioned IRIS; more than 800 GP practices nationally have had IRIS training, and over 5,000 women have been referred in to DVA support services by IRIS since 2010.

The cost-effectiveness of the IRIS trial was assessed using data from the trial and the programme was estimated to be good value for money (11). Given its national


For peer review only - http://bmjopen.bmj.com/site/about/guidelines.xhtml

implementation, IRIS became a real-life, long-term intervention, raising the need for a new economic evaluation outside the trial context. The aim of this study was to evaluate the cost-effectiveness of the IRIS programme now that it has been implemented across the UK. Our estimates use up-to-date figures from an MRC phase IV observational pragmatic implementation study (12) on costs and effectiveness from routine clinical practice and the most up-to-date model input parameters, including a recently updated Cochrane review of domestic violence advocacy (13).

## Methods

*Overview of economic evaluation*

This was a cost–utility analysis, comparing IRIS with usual care in general practices. The outcome measure was quality-adjusted life years (QALYs), as recommended for economic evaluations in the UK (14). The main analysis was from a societal perspective, as many of the costs of DVA are borne outside the health system; we also estimated cost utility from an NHS perspective. Costs were calculated in 2015/16 UK£. We calculated costs and benefits over a 10-year time horizon, with future costs and outcomes discounted at an annual rate of 3.5% (14).

*Model structure*

We developed a Markov model (Figure1) based on the previous analysis (11). The model has five states and the cycle length was six months; this length was chosen as it reflects the average amount of time women stay in contact with DVA advocacy services. We have used a half-cycle correction (15) A hypothetical cohort of 10,000 women aged 16 years or older was simulated moving between the states (Figure 1). Other than death, which is an absorbing state, women can transition between each of the other states 'Not abused', 'Abused but not

For peer review only


For peer review only - http://bmjopen.bmj.com/site/about/guidelines.xhtml

identified', 'Abused and identified, seeing advocate educator', 'Abuse and identified, not seeing advocate educator'. As the hypothetical cohort of women aged 16 or older were considered eligible for the intervention, all results were reported as “per woman aged 16 or older registered to GP practice”.

*Intervention*

The IRIS programme is a multi-component intervention that has been described in detail elsewhere (10, 11). In brief, it consists of two two-hour multidisciplinary training sessions, for the practice clinical team and one hour training for reception and ancillary staff. They are delivered jointly by an IRIS advocate educator from a local collaborating specialist DVA agency, alongside a clinician interested in DVA, the IRIS clinical lead. The advocate educator is central to the intervention, combining a training and support role to the practices with provision of advocacy to women referred. Other intervention components include a simple 4-question questionnaire addressing different aspects of DVA (Humiliation, Afraid, Raped and Kicked), the HARK template (16) in the electronic medical record triggered by entry of clinical problem codes (such as depression, anxiety, irritable bowel syndrome, pelvic pain and assault), an explicit referral pathway to a named IRIS advocate educator, and publicity materials about DVA visible in practices. Patients referred to the advocate educator are usually seen at the referring general practice, enhancing safety and confidentiality.

*Data collection and ethics approval*

Several different data sources were used in this study. Whenever possible, we have used observational data from the IRIS programme. These were collected by IRIS team members, liaising with advocacy agencies and local authorities. Given that we only use anonymized data, arising from the usual care of women, individual consent of women was not required.


For peer review only - http://bmjopen.bmj.com/site/about/guidelines.xhtml

This research project was given exemption from NHS Research Ethics processes, as it was classified as service evaluation. When observational data were unavailable, we have chosen to use peer-reviewed published data that was relevant to general practice and the UK. Each relevant parameter and its source are described in detail below.

*Prevalence of domestic abuse*

The proportion of women aged 16 years or older experiencing abuse was estimated based on published epidemiological data. This was taken from a cross sectional study carried out by Richardson and colleagues in east London (17), which reported a prevalence of 0.17 or 17% in the population of women consulting a general practitioner or practice nurse. This is an estimate of the prevalence of DVA in general practice, generalizable for England.

*Transition probabilities*

There are eight transitions between states in the model. Transition probabilities were obtained using observational data from the IRIS programme, the MOSAIC (MOthers' Advocates In the Community) programme (10, 18), the Office for National Statistics (19, 20) and Health & Social Care Information Centre (21), and a Cochrane review (13), evaluating the reduction of any type of domestic abuse with any type of advocacy. Observational data were obtained from commissioned IRIS sites that have been running for two years or more, where there was at least one full-time equivalent advocate educator and 20 general practices trained. It included 6 clinical commissioning groups (CCGs) in northern England, south-west England and London. Given the inclusion criteria, the sites represent the implementation of the programme. Table 1 provides the parameter values and their respective sources. Where no data were available, we have calculated estimates using the model calibration method described below.

*Model calibration*

Because of uncertainty surrounding transition probabilities from *Not abused* to *Abused but not identified* and *vice versa*, we used the prevalence of abuse (17%) estimated in Richardson and colleagues' study (17), to calibrate the model. The model was run for 3000 cycles, assuming that thereafter the number of women in each state would remain constant. This was based on our calculation of steady states. The transition probabilities from *Not abused* to *Abused but not identified* and *vice versa* were changed until the proportion of women in the *Not abused* state exactly reflected the observed prevalence (100-17=83%). The initial distribution of women in the three *Abused* states was also determined by this process.

*Utilities*

Each state in the Markov model was associated with a utility score, which consisted of a general measure of health-related quality-of-life (22), allowing us to measure QALYs associated with IRIS and the comparator based on the proportion of women in each health state in each of the 20 6-monthly cycles in the model, totalling 10 years. The utility score of women who were not abused was assumed to be 0.85 (23). Wittenberg and colleagues conducted a cross-sectional survey to estimate community preferences for health states resulting from intimate partner violence. Using a UK-based algorithm, they found the utility of women experiencing any abuse was 0.64. When the severity/frequency of violence was low, the mean utility was 0.65 and when the severity/frequency was moderate or severe the mean utility was 0.63. For women who were abused in our model, we assumed this was moderate to severe, giving a utility score of 0.63 (24). For women seeing an advocate educator, we used the utility value of women with low abuse (0.65), implying that seeing an

For peer review only - http://bmjopen.bmj.com/site/about/guidelines.xhtml

advocate educator slightly increased their quality-of-life scores. QALY gains were reported per woman aged 16 or older registered to GP practice.

*Costs*

We included: intervention costs, costs of onward referral, and costs associated with DVA (including costs to the UK National Health Service (NHS), lost economic output, costs to the criminal/civil justice system, and personal costs). Costs were also reported per woman aged 16 or older registered to GP practice.

One IRIS advocate educator typically provides training, support and advocacy services for 24 general practices at any one point in time. Intervention costs were calculated based on the actual budget of the IRIS programme in the six sites (including advocate educator salaries, travel, recruitment, laptop, telephone, publicity, clinician consultancy, evaluation and central management costs) at a total six month cost across all sites of £272,613. This was divided by the number of registered women aged 16+ in IRIS-trained general practices in these sites (n=595,902). Costs of onward referral from the advocate educator was based on the finding of contact time from the IRIS trial, in which an onward referral was given to 57% of women in contact with an advocate educator and 63% of these women accepted this referral. Therefore, although costs of onward referral were based on current budgets and salaries, the proportion of contact was obtained from the trial estimates. Total costs per onward referral were therefore £861. Taking into account the proportion of women given a referral and accepting it, and inflating it to 2015/16 UK£, average costs of advocate educator contact per abused woman were £312.

For peer review only

Costs associated with intimate partner violence in the UK are described by Walby and Olive (8). In their report, costs of lost economic output, health services, criminal justice system, civil justice system, social welfare, personal costs, specialised services and physical/emotional impact were individually reported, and total costs were €13,732 million (£11 billion) in 2012. We excluded costs of physical/emotional impact (€6,614 million), as they were not financial costs, but consisted of monetary valuing of health status, which in cost-effectiveness models ought to be captured in terms of QALYs; these were also not included in the original cost-effectiveness analysis. The remaining costs were converted to UK£ and inflated to 2015/16. Total costs per six months were £2,933 million. Based on the 2015 Crime Survey for England and Wales, it was estimated that 1.3 million women experienced intimate partner violence in 2015/16 in the UK (3). Mean costs per abused woman were therefore £2,043. We assumed that the costs of intimate partner abuse are similar to the costs of abuse by other family members, and that the costs would not differ between identified or unidentified abuse. In sensitivity analyses we have allowed the costs of identified abuse to increase or decrease by 10% compared to abuse that was not identified; similarly the costs of *Abused and identified, seeing advocate educator* were allowed to increase or decrease by 25%.

*Cost-utility analysis*

Costs and utilities were applied to each health state. Total costs and QALYs for the hypothetical cohort were generated for the IRIS programme and the control group. The main outcome was the incremental costs per QALY gained. In the UK an intervention is generally considered cost-effective when the incremental costs per QALY gained are less than £20,000 (14). We also presented the results of cost-effectiveness analysis in terms of incremental net monetary benefit (NMB). This was calculated as the mean incremental QALYs per woman



For peer review only - http://bmjopen.bmj.com/site/about/guidelines.xhtml

registered at the general practice accruing to IRIS multiplied by the decision-makers' maximum willingness to pay for a QALY (assumed to be £20,000), minus the mean incremental cost per woman. Negative incremental NMBs indicate that usual care was preferred on cost-effectiveness grounds and positive incremental NMBs favour IRIS.

The cost-utility analysis was conducted using pooled national data, but we have also evaluated the cost-effectiveness at different local sites. We allowed all parameters, including costs and benefits, to vary across sites and reported them individually.

*Sensitivity analysis*

All parameters were varied in a one-way sensitivity analysis, using lower and upper limits based on 95% uncertainty intervals. We undertook a probabilistic sensitivity analysis, drawing random samples from the probability distributions of all parameters in 1,000 simulations. All uncertainty intervals were calculated based on the 2.5$^{th}$ and 97.5$^{th}$ percentiles of the distribution of all the 1000 values in the probabilistic sensitivity analysis. The proportion of simulations with an incremental cost per QALY gained below the cost-effectiveness threshold was calculated for different values, ranging from £0 to £50,000. The results were presented in a cost-effectiveness acceptability curve.

*Patient and Public Involvement (PPI)*

We did not directly include PPI in this study, but the data collected from local IRIS Programmes was developed with PPI.

## Results

*Base case*

Parameter values used in the base case analysis are shown in Table 1, which also includes the parameters used in the original trial to allow for a direct comparison. The main differences between the parameters for this study and the trial parameters lie in the transition probabilities relating to the health state of 'abuse but not identified' and its cost.

Over the ten-year time horizon, mean total costs per woman were £4,416 in the intervention group, compared to £4,430 in the control group (Table 2(a)). The IRIS programme therefore saves £14 per woman aged 16 and older registered to GP practices, from a societal perspective over 10 years. Total QALYs per woman were 0.001 higher in the intervention group (6.671) than in the control group (6.669). Because the intervention was associated with lower costs and greater effectiveness the incremental cost per QALY gained was negative (i.e. IRIS dominates current practice as it is both cost-saving and more effective than usual care) and the incremental NMB was positive (£42). The incremental NMB was also positive (£22) when using an NHS-only perspective (Table 2(b)).

Table 2 also presents the results for each site. The table shows that IRIS dominated current practice, from a societal perspective, in sites 1, 2, 3 and 4, with an incremental net monetary benefit (NMB) of £41, £89, £29 and £59 respectively. From a NHS perspective, only in site 1 did IRIS dominate current practice, although it was cost-effective, using the threshold advised by NICE of £20,000 per QALY gained, in sites 2 (ICER £2,585 per QALY gained), 3 (ICER £3,055 per QALY gained) and 4 (ICER £8,317 per QALY gained). IRIS was found to be cost-effective (ICER £5,882 per QALY gained) and borderline cost-effective (ICER £21,229 per QALY gained) from a societal and NHS perspectives respectively in site 5, and it was not cost-effective from either perspective in site 6 (ICER £52,557 per QALY gained and ICER £64,427 per QALY gained respectively).


For peer review only - http://bmjopen.bmj.com/site/about/guidelines.xhtml

*Sensitivity analyses*

Across all sites combined, results were most sensitive to varying the transition probability from *Abused but not identified* to *Not abused*. When in the control arm this was varied from 0.049 to 0.051, the incremental NMB varied from £110 to -£26 (Figure 2). When it was varied similarly in the intervention arm, the incremental NMB varied from -£25 to £109. Figure 2 shows the 12 parameters that when varied had the highest impact on the incremental NMB.

Incremental costs and QALYs varied widely in probabilistic sensitivity analyses. The 95% uncertainty interval for incremental costs was -£151 to £37, for incremental QALYs it was -0.005 to 0.006 and for the incremental NMB it was -£247 to £351. Figure 3(a) shows a scatter plot of the incremental costs and incremental QALYs from the 1,000 simulations. The IRIS programme is cheaper and more effective than the absence of the programme (usual care), dominating current practice in 35% of the simulations and was dominated by the absence of the programme in 18% of the simulations. The IRIS programme was cost-effective in 61% of simulations when the cost-effectiveness threshold was £20,000 (Figure 3(b)).

## Discussion

*Summary*

We found that the IRIS GP training and service programme is likely to be cost-effective and cost-saving in the UK compared to usual care. The QALY gains associated with IRIS, which are average values for all eligible women aged 16 or over registered at a practice (and not, for example, those who have been abused), are small; these are balanced against an equally small incremental cost of the intervention. There is considerable uncertainty surrounding these results, but the probability that IRIS is cost-effective was more than 60% at the cost-

For peer review only

effectiveness threshold commonly used in the UK. The cost-effectiveness acceptability curve is relatively flat, implying that the results from IRIS do not change much regardless of the threshold used. In our view the shape of the CEAC is entirely consistent with the 95% uncertainty intervals. The fact that these values are close to 50% reflects there is a high level of uncertainty, and the fact that the probability that IRIS is cost-effective is just higher than 50% reflects the fact that IRIS is (slightly) favoured over the alternative according to our base case estimates. IRIS was more cost-effective when costs were measured from a societal perspective as the cost savings from reducing DVA were higher. IRIS was also cost-effective when taking an NHS-only perspective. There was some variation in value for money between sites, which appears to be driven mainly by the different rates of identification and/or referral, although different local costs have also contributed.

*Comparison with existing literature*

We contacted researchers in the field and searched the NHS Economic Evaluations Database and the HTA Database at the Centre for Reviews and Dissemination (25) for cost-effectiveness analyses of DVA programmes using the search terms “domestic violence” and “cost*” (28/08/2017). We identified four economic impact studies, all using modelling methods: one based on the pilot of the IRIS trial (22), another based on the main trial (11), the third based on an evaluation of independent domestic violence Advisors (IDVA) (26), and the fourth of a trial of cognitive trauma therapy for abused women who have left the abusive relationship (26). All the studies found the interventions cost-effective, despite uncertainty. Devine et al has reported a 75% probability of the DVA intervention being cost-effective (11), while Mallender et al reported 2 scenarios our of possible 5 in which the intervention is not cost-effective (26). Our findings are consistent with these previous studies. Our study is



For peer review only - http://bmjopen.bmj.com/site/about/guidelines.xhtml

the only one that analyses the economic impact of a primary care-based programme implemented outside of trial settings.

*Strengths and limitations*

Our analysis has the strength of being based on a previously published cost-effectiveness model, updated with real-life data. Importantly, intervention costs and the probability of referral with IRIS were based on actual clinical practice, rather than in a research setting. We also had new data for the probability of identifying abuse and for what happened to women who were abused in current practice without the programme. However, it was not possible to update all parameter values. In particular, we were unable to update the utility value estimates, although in the sensitivity analysis, we have allowed these to vary and results were relatively stable. Costs of the intervention were calculated by dividing the total costs of the programme over all registered women in practices with the IRIS programme. Many of these women will never experience abuse and therefore cannot directly benefit from the programme. If programme costs were divided over women experiencing abuse only, mean costs per woman would be higher. However, the QALYs gained would also be higher, as these are also calculated for all women in the practices rather than just those who were abused. In fact we have attempted to calculate these results dividing cost and QALYs over women experiencing abuse and the final ICER was unchanged, as both the numerator and denominator change by the same proportion. We did not include any impact of the IRIS programme on children exposed to DVA, as to our knowledge, there are no available cohort studies focusing on the cost and benefits of DVA interventions for this population which might mean that we have underestimated the programme's cost-effectiveness. This was also highlighted in the NICE economic analysis of interventions to reduce incidence and harm of


For peer review only - http://bmjopen.bmj.com/site/about/guidelines.xhtml

DVA: “It can be expected there are likely to be additional benefits such as [to] the children and wider family members of victims of domestic violence (p.11) (26).

Another limitation is that we have used mainly data on short-term outcomes, although modelled long-term outcomes. There is unfortunately little data on long-term outcomes of DVA and the effect of advocacy, although it is generally agreed that effects last for a long time. This, however, bias our estimates against the intervention, implying our results are conservative.

*Implications for research and/or practice*

The IRIS programme is likely to be cost-effective and cost-saving when implemented in the real life of the in the UK National Health System. In order to decrease uncertainty around the cost-effectiveness estimates of IRIS and programmes like it, more data are needed on the utilities of women identified and women seeing an advocate and on long-term outcomes associated with DVA. Furthermore, future research should endeavour to understand the impacts and economic burden of DVA on exposed children, other family members and friends, as well as focus on collecting up-to-date utility values for women subject to DVA in each health state.

Finally, our study has shown that there is moderate variation in the value for money of IRIS across different sites, implying qualitative research could focus on identifying the causes of such variation, in order to reduce it.



For peer review only - http://bmjopen.bmj.com/site/about/guidelines.xhtml

**Authors' Contribution**

SM, CG, SE, AS and GF have designed the study. EB, TV, SM, FS and AD have developed the Markov model and carried out the analysis of data. AS, FES, SD, CR, NL and MJ have collected and validated the data. EB and SM have produced the initial draft. All authors have critically revised the manuscript and approved the final version.

**Competing Interests disclosure**

MJ has been paid by the IRIS project since 2007 for employment as an IRIS Advocate Educator and then as a National Implementation Manager. She is currently paid by IRISi, a social enterprise that is promoting the commissioning of the IRIS programme, for employment as Chief Executive. GF reports grants from National Institute for Health Research (NIHR), during the conduct of the study; and he is a non-executive board member of IRISi. All other authors disclose no competing interests.

**Funding and Acknowledgements**

This research was funded by the National Institute for Health Research (NIHR) Collaboration for Leadership in Applied Health Research and Care North Thames at Barts Health NHS Trust.

GF acknowledges the support of the National Institute for Health Research from a programme grant for applied research RP-PG-0614-20012 REPROVIDE (Reaching Everyone Programme of Research on Violence in diverse Domestic Environments).

The views expressed are those of the author(s) and not necessarily those of the NHS, the NIHR or the Department of Health.

For peer review only

We would like to thank our IRIS partners who deliver the programme in the sites, especially those in northern England, south-west England and London who took the time and effort to provide us with data.

**Data sharing**

The anonymised data used in this study can be obtained from the corresponding author.

For peer review only

## References

1. Assembly UG. Declaration on the Elimination of Violence against Women. UN General Assembly. 1993.

2. Garcia-Moreno C, Jansen HA, Ellsberg M, Heise L, Watts CH. Prevalence of intimate partner violence: findings from the WHO multi-country study on women's health and domestic violence. Lancet. 2006;368(9543):1260-9.

3. ONS. Intimate personal violence and partner abuse compendium. 2016.

4. Bonomi AE, Anderson ML, Reid RJ, Rivara FP, Carrell D, Thompson RS. Medical and psychosocial diagnoses in women with a history of intimate partner violence. Archives of internal medicine. 2009;169(18):1692-7.

5. Campbell JC. Health consequences of intimate partner violence. Lancet. 2002;359(9314):1331-6.

6. Tollestrup K, Sklar D, Frost FJ, Olson L, Weybright J, Sandvig J, et al. Health indicators and intimate partner violence among women who are members of a managed care organization. Prev Med. 1999;29(5):431-40.

7. Coid J, Petruckevitch A, Chung WS, Richardson J, Moorey S, Feder G. Abusive experiences and psychiatric morbidity in women primary care attenders. The British journal of psychiatry : the journal of mental science. 2003;183:332-9; discussion 40-1.

8. Walby S, Olive P. Estimating the costs of gender-based violence in the European Union. European Institute for Gender Equality; 2014.

9. European Innovation Partnership on A, Healthy Ageing APB, Mechanisms of the Development of Allergy WP, Global Alliance against Chronic Respiratory D, Bousquet J, Addis A, et al. Integrated care pathways for airway diseases (AIRWAYS-ICPs). The European respiratory journal. 2014;44(2):304-23.

10. Feder G, Davies RA, Baird K, Dunne D, Eldridge S, Griffiths C, et al. Identification and Referral to Improve Safety (IRIS) of women experiencing domestic violence with a primary care training and support programme: a cluster randomised controlled trial. Lancet. 2011;378(9805):1788-95.

11. Devine A, Spencer A, Eldridge S, Norman R, Feder G. Cost-effectiveness of Identification and Referral to Improve Safety (IRIS), a domestic violence training and support programme for primary care: a modelling study based on a randomised controlled trial. BMJ open. 2012;2(3).

12. Sohal A, Dowrick A, El-Shoghri F, Beresford L, Lewis N, Barbosa E, et al. Improving the healthcare response to domestic violence and abuse in primary care: protocol for

For peer review only

evaluation of a complex intervention's implementation into multiple general practices, including a phase IV observational segmented regression interrupted time series analysis BMJ Public Health. 2018 forthcoming.

13. Rivas C, Ramsay J, Sadowski L, Davidson LL, Dunne D, Eldridge S, et al. Advocacy interventions to reduce or eliminate violence and promote the physical and psychosocial well-being of women who experience intimate partner abuse. status and date: New search for studies and content updated (no change to conclusions), published in. 2015(12).

14. NICE NIoHaCE. Guide to the methods of technology appraisal 2013 [Available from: http://www.nice.org.uk/media/D45/1E/GuideToMethodsTechnologyAppraisal2013.pdf.

15. Naimark DM, Bott M, Krahn M. The half-cycle correction explained: two alternative pedagogical approaches. Medical Decision Making. 2008;28(5):706-12.

16. Sohal H, Eldridge S, Feder G. The sensitivity and specificity of four questions (HARK) to identify intimate partner violence: a diagnostic accuracy study in general practice. BMC family practice. 2007;8(1):49.

17. Richardson J, Coid J, Petruckevitch A, Chung WS, Moorey S, Feder G. Identifying domestic violence: cross sectional study in primary care. Bmj. 2002;324(7332):274.

18. Taft AJ, Small R, Hegarty KL, Watson LF, Gold L, Lumley JA. Mothers' AdvocateS In the Community (MOSAIC)--non-professional mentor support to reduce intimate partner violence and depression in mothers: a cluster randomised trial in primary care. BMC public health. 2011;11:178.

19. Statistics OoN. Crime Statistics, Focus on Violent Crime and Sexual Offences 2013/14 [Available from: http://www.ons.gov.uk/ons/rel/crime-stats/crime-statistics/focus-on-violent-crime-and-sexual-offences--2013-14/index.html.

20. Statistics OfN. Mortality Statistics: Deaths Registered in England and Wales 2013 [Available from: http://www.ons.gov.uk/ons/publications/re-reference-tables.html?edition=tcm%3A77-327590.

21. Centre HSCI. Numbers of Patients Registered at a GP practice January 2014

22. Horsman J, Furlong W, Feeny D, Torrance G. The Health Utilities Index (HUI®): concepts, measurement properties and applications. Health and quality of life outcomes. 2003;1(1):54.

23. Kind K, Hardman G, Macran S. UK Population norms for EQ-5D. University of York; 1999.

24. Wittenberg E, Lichter EL, Ganz ML, McCloskey LA. Community preferences for health states associated with intimate partner violence. Medical care. 2006;44(8):738-44.

For peer review only

25. York Uo. Centre for Reviews and Dissemination [Available from: http://www.crd.york.ac.uk/CRDWeb/HomePage.asp.

26. Mallender J, Venkatachalam M, Onwude O, Jhita T. Economic analysis of interventions to reduce incidence and harm of domestic violence. London: National Institute for Health and Care Excellence. 2013.

For peer review only

**Table 1. Model input parameters: probabilities; utilities; and, costs.**

| Parameter | Base case value | Lower limit | Upper limit | Distribution | Source | IRIS trial base value[1] |
|---|---|---|---|---|---|---|
| ***Probabilities*** | | | | | | |
| Proportion of women experiencing abuse | 0.17 | 0.147 | 0.194 | Beta | (17) | 0.17 |
| *Starting distribution for women who are abused* | | | | | | |
| Abused and identified, seeing advocate educator | 0.003¶ | 0 | 0.0066 | Uniform | * | - |
| Abused and identified, not seeing advocate educator | 0.033¶ | 0 | 0.0660 | Uniform | * | - |
| Abused but not identified | 0.964¶ | - | - | Uniform | Complement | - |
| *Transition probabilities* | | | | | | |
| Not abused to Abused but not identified | 0.0037¶ | 0.0004 | 0.0106 | Dirichlet | * | 0.0075 |
| Not abused to Dead | 0.00551¶ | 0.0010 | 0.0136 | Dirichlet | (13, 15) | 0.0058 |
| Stay in Not abused | 0.9908¶ | - | - | Dirichlet | Complement | 0.9867 |
| Abused but not identified to Not abused (control) | 0.0500¶ | 0.0450 | 0.0553 | Dirichlet | * | 0.025 |
| Abused but not identified to Abused and identified, not seeing advocate educator (control) | 0.0027¶ | 0.0016 | 0.0040 | Dirichlet | IRIS-programme local sites | 0.0094 |



For peer review only - http://bmjopen.bmj.com/site/about/guidelines.xhtml

| | | | | | | |
|---|---|---|---|---|---|---|
| Abused but not identified to Abused and identified, seeing advocate educator (control) | 0.0005¶ | 0.0001 | 0.0011 | Dirichlet | IRIS-programme local sites | 0.0016 |
| Abused but not identified to Dead (control) | 0.00554¶ | 0.0039 | 0.0074 | Dirichlet | (13, 15) | 0.0059 |
| Stay in Abused but not identified (control) | 0.9444¶ | - | - | Dirichlet | Complement | 0.9581 |
| Abused but not identified to Not abused (intervention) | 0.0500¶ | 0.0450 | 0.0553 | Dirichlet | * | 0.025 |
| Abused but not identified to Abused and identified, not seeing advocate educator (intervention) | 0.0109¶ | 0.0086 | 0.0135 | Dirichlet | IRIS-programme local sites | 0.0207 |
| Abused but not identified to Abused and identified, seeing advocate educator (intervention) | 0.0056¶ | 0.0040 | 0.0076 | Dirichlet | IRIS-programme local sites | 0.0101 |
| Abused but not identified to Dead (intervention) | 0.00554¶ | 0.0039 | 0.0074 | Dirichlet | (6) | 0.0059 |
| Stay in Abused but not identified (intervention) | 0.9419 ¶ | - | - | Dirichlet | Complement | 0.9383 |
| Abused and identified, seeing advocate educator to Not abused | 0.1408¶ | 0.0707 | 0.2301 | Dirichlet | (18) | 0.0888 |
| Abused and identified, seeing advocate educator to Dead | 0.00554¶ | 0.0000 | 0.0309 | Dirichlet | (13, 15) | 0.0059 |
| Stay in Abused and identified, seeing advocate educator | 0.8536¶ | - | - | Dirichlet | Complement | 0.9053 |



For peer review only - http://bmjopen.bmj.com/site/about/guidelines.xhtml

| | | | | | | |
|---|---|---|---|---|---|---|
| Abused and identified, not seeing advocate educator to Not abused | 0.0781¶ | 0.0136 | 0.1912 | Dirichlet | (18) | 0.0717 |
| Abused and identified, not seeing advocate educator to Dead | 0.00554¶ | 0.0000 | 0.0438 | Dirichlet | (13, 15) | 0.0059 |
| Stay in Abused and identified, not seeing advocate educator | 0.9163¶ | - | - | Dirichlet | Complement | 0.9223 |
| ***Utilities*** | | | | | | |
| Not abused | 0.85 | 0.840 | 0.860 | Beta | (23) | - |
| Abused but not identified | 0.63 | 0.503 | 0.749 | Beta | (24) | - |
| Abused and identified, seeing advocate educator | 0.65 | 0.518 | 0.771 | Beta | (24) | - |
| Abused and identified, not seeing advocate educator | 0.63 | 0.503 | 0.749 | Beta | (24) | - |
| ***Costs*** | | | | | | |
| Costs of the intervention, per women registered, per 6 months | £0.46¶ | £0.01 | £1.69 | Gamma | IRIS-programme local sites | £0.55 |
| Cost of onward referral, once | £312¶ | £8 | £1127 | Gamma | IRIS-programme | £298 |



For peer review only - http://bmjopen.bmj.com/site/about/guidelines.xhtml

| | | | | | | |
|---|---|---|---|---|---|---|
| | | | | | local sites & (11) | |
| Cost of Abused but not identified | £2043 | £52 | £7536 | Gamma | (8) | £4721 |
| Weighted costs Abused and identified, seeing advocate educator | 1 | 0.75 | 1.25 | Gamma | Assumption | - |
| Weighted costs Abused and identified, not seeing advocate educator | 1 | 0.9 | 1.1 | Gamma | Assumption | - |

Costs are in 2015/16 UK£.
* Internal calculation based on model calibration.
¶ Value updated from Devine et al (11).
[1] Values obtained from Devine et al (11).



For peer review only - http://bmjopen.bmj.com/site/about/guidelines.xhtml

**Table 2. Base case results.**

| | (a) Societal perspective | | | (b) NHS-only perspective | | |
|---|---|---|---|---|---|---|
| **National IRIS (pooled results)** | **Costs** | **QALYs** | **Cost-effectiveness** | **Costs** | **QALYs** | **Cost-effectiveness** |
| Intervention (IRIS programme) | £4416 | 6.671 | | £1238 | 6. 671 | |
| Control (no programme) | £4430 | 6.669 | | £1232 | 6. 669 | |
| Difference (intervention vs. control) | -£14 | 0.001 | -ve (intervention dominates control) | £6 | 0.001 | £3913 per QALY gained |
| Incremental NMB* | | | £42 | | | £22 |
| **Local site 1** | | | | | | |
| Intervention (IRIS programme) | £4318 | 6.671 | | £1231 | 6.671 | |
| Control (no programme) | £4334 | 6.669 | | £1232 | 6.669 | |
| Difference (intervention vs. control) | -£16 | 0.001 | -ve (intervention dominates control) | -£1 | 0.001 | -ve (intervention dominates control) |
| Incremental NMB* | | | £41 | | | £26 |
| **Local site 2** | | | | | | |
| Intervention (IRIS programme) | £4305 | 6.673 | | £1240 | 6.673 | |
| Control (no programme) | £4333 | 6.670 | | £1232 | 6.670 | |
| Difference (intervention vs. control) | -£28 | 0.003 | -ve (intervention | £8 | 0.003 | £2585 per QALY |



For peer review only - http://bmjopen.bmj.com/site/about/guidelines.xhtml

| | | | | | | |
|---|---|---|---|---|---|---|
| | | | dominates control) | | | gained |
| Incremental NMB* | | | £89 | | | £54 |
| **Local site 3** | | | | | | |
| Intervention (IRIS programme) | £4325 | 6.671 | | £1235 | 6.671 | |
| Control (no programme) | £4334 | 6.670 | | £1232 | 6.670 | |
| Difference (intervention vs. control) | -£9 | 0.001 | -ve (intervention dominates control) | £3 | 0.001 | £3055 per QALY gained |
| Incremental NMB* | | | £29 | | | £17 |
| **Local site 4** | | | | | | |
| Intervention (IRIS programme) | £4326 | 6.672 | | £1253 | 6.672 | |
| Control (no programme) | £4334 | 6.669 | | £1232 | 6.669 | |
| Difference (intervention vs. control) | -£8 | 0.003 | -ve (intervention dominates control) | £21 | 0.003 | £8317 per QALY gained |
| Incremental NMB* | | | £59 | | | £30 |
| **Local site 5** | | | | | | |
| Intervention (IRIS programme) | £4337 | 6.670 | | £1244 | 6.670 | |
| Control (no programme) | £4332 | 6.669 | | £1232 | 6.669 | |
| Difference (intervention vs. control) | £4 | 0.001 | £5882 per QALY | £12 | 0.001 | £21229 per QALY |



For peer review only - http://bmjopen.bmj.com/site/about/guidelines.xhtml

| | | | | | | |
|---|---|---|---|---|---|---|
| | | | gained | | | gained |
| Incremental NMB* | | | £6 | | | £0 |
| **Local site 6** | | | | | | |
| Intervention (IRIS programme) | £4395 | 6.671 | | £1307 | 6.671 | |
| Control (no programme) | £4334 | 6.670 | | £1232 | 6.670 | |
| Difference (intervention vs. control) | £61 | 0.001 | £52557 per QALY gained | £75 | 0.001 | £64427 per QALY gained |
| Incremental NMB* | | | -£38 | | | -£52 |

NMB = net monetary benefit. QALY = quality-adjusted life year. Costs are in 2015/16 UK£. Numbers may not sum due to rounding.

*Measured at a willingness to pay for a QALY of £20 000.



For peer review only - http://bmjopen.bmj.com/site/about/guidelines.xhtml

**Figure 1. Health states and movement between health states in Markov model.**

Legend: The model starts with all women in either the ‘Not abused’ state or one of the states associated with abuse, based on the prevalence of DVA (see text). Women in the ‘Not abused’ state could stay in this state, move to ‘Abused but not identified’ or die from any cause. Once women were in the ‘Abused but not unidentified’ state, they could stay in that state, move back to ‘Not abused’, move to ‘Abused and identified, seeing advocate’ or ‘Abused and identified, not seeing advocate’ or die. Women in the ‘Abused and identified’ states could stay in these states, move back to ‘Not abused’ or die.

For peer review only



For peer review only - http://bmjopen.bmj.com/site/about/guidelines.xhtml

**Figure 2. Univariate sensitivity analysis.**

Legend: All analyses are as for the base case analysis with univariate adjustment of the parameters listed (see text). Results are point estimates of the incremental net monetary benefit (NMB) of the intervention vs. control. The incremental net monetary benefit is calculated at a maximum willingness to pay for a QALY of £20 000.

For peer review only



For peer review only - http://bmjopen.bmj.com/site/about/guidelines.xhtml

**Figure 3. Probabilistic sensitivity analysis.**

(a) **Scatter plot of incremental costs and incremental QALYs from 1000 simulations**
(b) **Cost-effectiveness acceptability curve showing the probability in percentage terms that the intervention is cost-effective vs. control at different values of the maximum willingness to pay for a QALY**

Legend: QALY = quality-adjusted life year. Costs are in 2015/16 UK£.

For peer review only



For peer review only - http://bmjopen.bmj.com/site/about/guidelines.xhtml

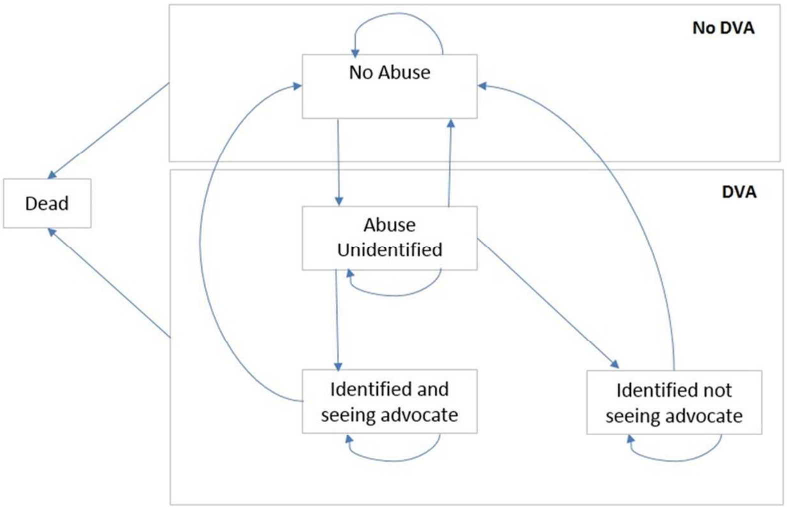


Figure 1. Health states and movement between health states in Markov model.
Legend: The model starts with all women in either the 'Not abused' state or one of the states associated with abuse, based on the prevalence of DVA (see text). Women in the 'Not abused' state could stay in this state, move to 'Abused but not identified' or die from any cause. Once women were in the 'Abused but not unidentified' state, they could stay in that state, move back to 'Not abused', move to 'Abused and identified, seeing advocate' or 'Abused and identified, not seeing advocate' or die. Women in the 'Abused and identified' states could stay in these states, move back to 'Not abused' or die.

160x90mm (300 x 300 DPI)

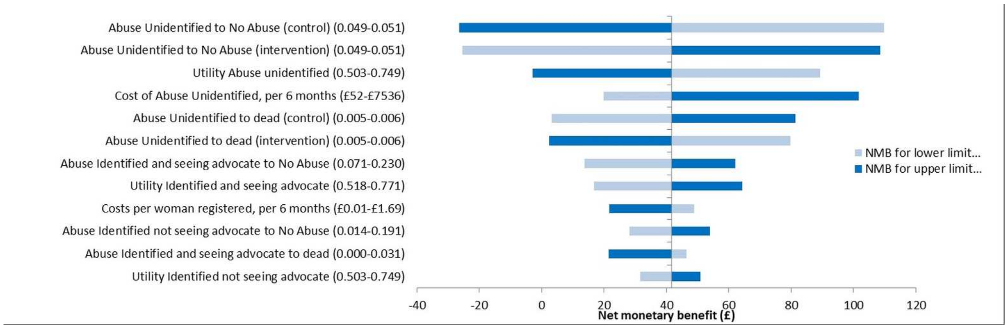


Figure 2. Univariate sensitivity analysis.
Legend: All analyses are as for the base case analysis with univariate adjustment of the parameters listed (see text). Results are point estimates of the incremental net monetary benefit (NMB) of the intervention vs. control. The incremental net monetary benefit is calculated at a maximum willingness to pay for a QALY of £20 000.

280x90mm (300 x 300 DPI)

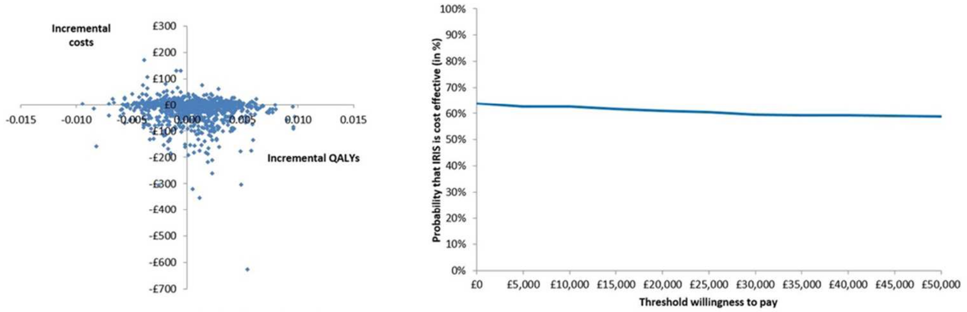


Figure 3. Probabilistic sensitivity analysis.
(a) Scatter plot of incremental costs and incremental QALYs from 1000 simulations
(b) Cost-effectiveness acceptability curve showing the probability in percentage terms that the intervention is cost-effective vs. control at different values of the maximum willingness to pay for a QALY
Legend: QALY = quality-adjusted life year. Costs are in 2015/16 UK£.

90x28mm (300 x 300 DPI)

For peer review only

## CHEERS checklist—Items to include when reporting economic evaluations of health interventions

| Section/item | Item No | Recommendation | Reported on page No/ line No |
|---|---|---|---|
| **Title and abstract** | | | |
| Title | 1 | Identify the study as an economic evaluation or use more specific terms such as "cost-effectiveness analysis", and describe the interventions compared. | page 1, line 1 to 4 |
| Abstract | 2 | Provide a structured summary of objectives, perspective, setting, methods (including study design and inputs), results (including base case and uncertainty analyses), and conclusions. | page 5, line 1 to page 6, line 12 |
| **Introduction** | | | |
| Background and objectives | 3 | Provide an explicit statement of the broader context for the study. | page 7, line 2 to 25 |
| | | Present the study question and its relevance for health policy or practice decisions. | page 7, line 25 to page 8, line 6 |
| **Methods** | | | |
| Target population and subgroups | 4 | Describe characteristics of the base case population and subgroups analysed, including why they were chosen. | page 8, line 22 to page 9, line 3; page 10, line 5 to 22 |
| Setting and location | 5 | State relevant aspects of the system(s) in which the decision(s) need(s) to be made. | page 10, line 17 to 20 |
| Study perspective | 6 | Describe the perspective of the study and relate this to the costs being evaluated. | page 8, line 12 to 14; page 29 to 31, table 2 |
| Comparators | 7 | Describe the interventions or strategies being compared and state why they were chosen. | page 8, line 10 |
| Time horizon | 8 | State the time horizon(s) over which costs and consequences are being evaluated and say why appropriate. | page 8, line 14 to 15 |
| Discount rate | 9 | Report the choice of discount rate(s) used for costs and outcomes and say why appropriate. | page 8, line 15 to 16 |
| Choice of health outcomes | 10 | Describe what outcomes were used as the measure(s) of benefit in the evaluation and their relevance for the type of analysis performed. | page 11, line 10 to 23 |
| Measurement of effectiveness | 11a | *Single study-based estimates:* Describe fully the design features of the single effectiveness study and why the single study was a sufficient source of clinical effectiveness data. | page 9, line 20 to page 10, line 3; page 10, line 5 to 22; page 11, line 1 to 8; pages 25 to 27, table 1 |
| | 11b | *Synthesis-based estimates*: Describe fully the methods used for identification of included studies and synthesis of clinical effectiveness data. | |
| Measurement and valuation of preference based outcomes | 12 | If applicable, describe the population and methods used to elicit preferences for outcomes. | not applicable |
| Estimating resources and costs | 13a | *Single study-based economic evaluation:* Describe approaches used to estimate resource use associated with the alternative interventions. Describe primary or secondary research methods for valuing each resource item in terms of its unit cost. Describe any adjustments made to approximate to opportunity costs. | |

For peer review only

| Section/item | Item No | Recommendation | Reported on page No/ line No |
|---|---|---|---|
| | 13b | *Model-based economic evaluation:* Describe approaches and data sources used to estimate resource use associated with model health states. Describe primary or secondary research methods for valuing each resource item in terms of its unit cost. Describe any adjustments made to approximate to opportunity costs. | page 9, line 20 to page 10, line 3; page 12, line 1 to page 13, line 11; pages 27 and 28, table 1 |
| Currency, price date, and conversion | 14 | Report the dates of the estimated resource quantities and unit costs. Describe methods for adjusting estimated unit costs to the year of reported costs if necessary. Describe methods for converting costs into a common currency base and the exchange rate. | page 28, table 1; page 8, line 14 |
| Choice of model | 15 | Describe and give reasons for the specific type of decision-analytical model used. Providing a figure to show model structure is strongly recommended. | page 8, line 18 to 25; figure 1 |
| Assumptions | 16 | Describe all structural or other assumptions underpinning the decision-analytical model. | page 11, line 14 to 23; page 10, line 25 to page 11, line 8; |
| Analytical methods | 17 | Describe all analytical methods supporting the evaluation. This could include methods for dealing with skewed, missing, or censored data; extrapolation methods; methods for pooling data; approaches to validate or make adjustments (such as half cycle corrections) to a model; and methods for handling population heterogeneity and uncertainty. | page 8, line 21 to 22; page 10, line 25 to page 11, line 8; |
| **Results** | | | |
| Study parameters | 18 | Report the values, ranges, references, and, if used, probability distributions for all parameters. Report reasons or sources for distributions used to represent uncertainty where appropriate. Providing a table to show the input values is strongly recommended. | page 14, line 17 to 20; page 25 to 28, table 1 |
| Incremental costs and outcomes | 19 | For each intervention, report mean values for the main categories of estimated costs and outcomes of interest, as well as mean differences between the comparator groups. If applicable, report incremental cost-effectiveness ratios. | page 14, line 21 to page 15, line 4; page 29 to 31, table 2; |
| Characterising uncertainty | 20a | *Single study-based economic evaluation:*Describe the effects of sampling uncertainty for the estimated incremental cost and incremental effectiveness parameters, together with the impact of methodological assumptions (such as discount rate, study perspective). | |
| | 20b | *Model-based economic evaluation:* Describe the effects on the results of uncertainty for all input parameters, and uncertainty related to the structure of the model and assumptions. | page 15, line 18 to page 16, line 6; figure 2; figure 3a and 3b |
| Characterising heterogeneity | 21 | If applicable, report differences in costs, outcomes, or cost-effectiveness that can be explained by variations between subgroups of patients with different baseline characteristics or other observed variability in effects that are not reducible by more information. | Page 15, line 6 to 15 |
| **6 Discussion** | | | |
| Study findings, limitations, | 22 | Summarise key study findings and describe how they support the conclusions reached. Discuss limitations | page 16, line 9 to page 19, line 5 |

For peer review only

| Section/item | Item No | Recommendation | Reported on page No/ line No |
|---|---|---|---|
| generalisability, and current knowledge | | and the generalisability of the findings and how the findings fit with current knowledge. | |
| **Other** | | | |
| Source of funding | 23 | Describe how the study was funded and the role of the funder in the identification, design, conduct, and reporting of the analysis. Describe other non-monetary sources of support. | page 20, line 14 to 20 |
| Conflicts of interest | 24 | Describe any potential for conflict of interest of study contributors in accordance with journal policy. In the absence of a journal policy, we recommend authors comply with International Committee of Medical Journal Editors recommendations. | page 20, line 7 a 12 |

For consistency, the CHEERS statement checklist format is based on the format of the CONSORT statement checklist

For peer review only

BMJ Open

# Cost-effectiveness of a domestic violence and abuse training and support programme in primary care in the real world: updated modelling based on a MRC phase IV observational pragmatic implementation study



| | |
|---|---|
| Journal: | *BMJ Open* |
| Manuscript ID | bmjopen-2017-021256.R2 |
| Article Type: | Research |
| Date Submitted by the Author: | 03-Jul-2018 |



| | |
|---|---|
| Complete List of Authors: | Capelas Barbosa, Estela; University College London, Department of Applied Health Research<br>Verhoef, Talitha; University College London, Departmet of Applied Health Research<br>Morris, Stephen; University College London, Department of Applied Health Research<br>Solmi, Francesca; University College London, Division of Psychiatry; University College London<br>Johnson, Medina; IRISi<br>Sohal, Alex; Queen Mary University of London, Centre for Primary Care and Public Health<br>El-Shogri, Farah; Queen Mary University of London<br>Dowrick, Susanna; Queen Mary University of London, Centre for Primary Care and Public Health<br>Ronalds, Clare; Manchester Women's Aid, Pankhurst Trust Incorporating<br>Griffiths, Chris; Barts and The London School of Medicine and Dentistry,<br>Eldridge, Sandra; Queen Mary University of London, Centre for Primary Care and Public Health<br>Lewis, Natalia; Queen Mary University of London, Centre for Primary Care and Public Health<br>Devine, A; University of Oxford , Centre forTropical Medicine and Global Health; Mahidol University Phayathai Campus, Mahidol-Oxford Tropical Medicine Research Unit<br>Spencer, Anne; University of Exeter Medical School<br>Feder, Gene; University of Bristol, Community based medicine |



| | |
|---|---|
| <b>Primary Subject Heading</b>: | Health economics |
| Secondary Subject Heading: | General practice / Family practice |
| Keywords: | Domestic violence, intimate partner violence, training programme, general practice, family medicine primary care, cost-effectiveness |
| | |

SCHOLARONE™
Manuscripts

For peer review only

| | |
|---|---|
| **Title** | **Cost-effectiveness of a domestic violence and abuse training and support programme in primary care in the real world: updated modelling based on a MRC phase IV observational pragmatic implementation study** |

Estela C. Barbosa (Corresponding author)
Department of Applied Health Research, UCL
Gower Street, London, UK WC1E 6BT
020 3108 3024
Email: e.barbosa@ucl.ac.uk

Talitha I. Verhoef
Department of Applied Health Research, UCL
1-19 Torrington Pl, London WC1E 7HB
United Kingdom

Steve Morris
Department of Applied Health Research, UCL
Gower Street, London, UK WC1E 6BT
020 3108 3024

Francesca Solmi
Division of Psychiatry, UCL
149 Tottenham Court Road, London W1T 7NF
United Kingdom

Medina Johnson
IRISi Interventions
PO Box 174 | Chepstow | NP16 9BT
United Kingdom

For peer review only

Alex Sohal
NIHR CLAHRC North Thames at Bart's Health NHS Trust, Centre for Primary Care and Public Health, Queen Mary University of London
58 Turner St, London,
United Kingdom EC1 5AT

Farah El-Shoghri
NIHR CLAHRC North Thames at Bart's Health NHS Trust, Centre for Primary Care and Public Health, Queen Mary University of London
58 Turner St, London,
United Kingdom EC1 5AT

Susanna Dowrick
NIHR CLAHRC North Thames at Bart's Health NHS Trust, Centre for Primary Care and Public Health, Queen Mary University of London
58 Turner St, London,
United Kingdom EC1 5AT

Clare Ronalds
IRIS GP Project Lead Manchester, Pankhurst Trust Incorporating Manchester Women's Aid
60-62 Nelson Street, Manchester M13 9WP
United Kingdom

Chris Griffiths
NIHR CLAHRC North Thames at Bart's Health NHS Trust, Centre for Primary Care and Public Health, Queen Mary University of London
58 Turner St, London,
United Kingdom EC1 5AT

For peer review only

Sandra Eldridge
NIHR CLAHRC North Thames at Bart's Health NHS Trust, Centre for Primary Care and Public Health, Queen Mary University of London

58 Turner St, London,

United Kingdom EC1 5AT

Natalia V. Lewis
NIHR CLAHRC North Thames at Bart's Health NHS Trust, Centre for Primary Care and Public Health, Queen Mary University of London

58 Turner St, London,

United Kingdom EC1 5AT

Angela Devine
Mahidol-Oxford Tropical Medicine Research Unit, Mahidol University and Centre for Tropical Medicine and Global Health, Nuffield Department of Clinical Medicine, University of Oxford

Anne Spencer
University of Exeter Medical School

St Luke's Campus

Heavitree Road, Exeter EX1 2LU

United Kingdom

Gene Feder
School of Social and Community Medicine, University of Bristol

39 Whatley Road, Bristol BS8 2PS

United Kingdom

For peer review only

Key words: Domestic violence, intimate partner violence, training programme, general practice, family medicine primary care, cost-effectiveness.

Word count: Abstract: 300

4118 (excluding title page, abstract, tables and acknowledgements and references)

For peer review only

**Abstract**

*Objectives*:

To evaluate the cost-effectiveness of the implementation of the Identification and Referral to Improve Safety (IRIS) programme using up-to-date real-world information on costs and effectiveness from routine clinical practice. A Markov model was constructed to estimate mean costs and quality-adjusted life-years (QALYs) of IRIS versus usual care per woman registered at a general practice from a societal and health service perspective with a ten-year time horizon.

*Design and Setting*:

Cost–utility analysis in UK general practices, including data from six sites which have been running IRIS for at least two years across England.

*Participants*:

Based on the Markov model, which uses health states to represent possible outcomes of the intervention, we stipulated a hypothetical cohort of 10,000 women aged 16 years or older.

*Interventions*

The IRIS trial was a randomised controlled trial that tested the effectiveness of a primary care training and support intervention to improve the response to women experiencing DVA, and found it to be cost-effective. As a result, the IRIS programme has been implemented across the UK, generating data on costs and effectiveness outside a trial context.

*Results*:

The IRIS programme saved £14 per woman aged 16 or older registered in general practice (95% uncertainty interval [-£151; £37]) and produced QALY gains of 0.001 per woman (95% uncertainty interval [-0.005; 0.006]). The incremental net monetary benefit was positive both from a societal and NHS perspective (£42 and £22 respectively) and the IRIS programme was

For peer review only

cost-effective in 61% of simulations using real life data when the cost-effectiveness threshold was £20 000 per QALY gained as advised by NICE.

*Conclusion*:

The IRIS programme is likely to be cost-effective and cost-saving from a societal perspective in the UK and cost effective from a health service perspective, though there is considerable uncertainty surrounding these results, reflected in the large uncertainty intervals.

**Strengths and limitations of this study**

- We have used up-to-date routine data from several sites across England to evaluate the value for money of IRIS, a domestic violence training programme.
- We were unable to include any impact of the IRIS programme on children exposed to DVA, as to our knowledge, there are no available cohort studies focusing on the cost and benefits of DVA interventions for this population.
- We have used mainly data on short-term outcomes, although modelled long-term outcomes, as to our knowledge, no study has tracked women subject to DVA over long periods of time.

For peer review only

## Introduction

The lifetime prevalence of domestic violence and abuse (DVA) against women, including any form of controlling, coercive, threatening behaviour, violence and abuse, as well as non-physical forms of abuse as defined by the United Nations (1), varies internationally from 15% to 71% (2). In the United Kingdom, in the year ending March 2017, 7.5% of women (1.2 million) experienced domestic abuse (3). Women who experience DVA suffer chronic health problems including gynaecological problems, gastrointestinal disorders, neurological symptoms, chronic pain, cardiovascular conditions and mental health problems (4-7). In 2012, the cost of DVA in the UK, including medical and social services, lost economic output and emotional costs, was estimated to be £11 billion (8). While such estimates highlight the importance of DVA as a public health and clinical problem, information on cost-effectiveness is needed to make an economic case for investment in DVA interventions in health care, particularly when health systems are dominated by austerity.

The Identification and Referral to Improve Safety (9) trial tested the effectiveness of a training and support intervention for general practice teams in two English cities (10). Discussions about DVA between clinicians and patients were 22 times greater in the intervention practices compared with the control practices. Primary care practices that delivered the intervention also experienced a 6 fold and 3 fold increase in referrals received by DVA agencies and DVA-related notes in the patient medical records, respectively. The IRIS programme can now be commissioned across the UK: as of December 2016, 34 UK areas had commissioned IRIS; more than 800 GP practices nationally have had IRIS training, and over 5,000 women have been referred in to DVA support services by IRIS since 2010.

For peer review only - http://bmjopen.bmj.com/site/about/guidelines.xhtml

The cost-effectiveness of the IRIS trial was assessed using data from the trial and the programme was estimated to be good value for money (11). Given its national implementation, IRIS became a real-life, long-term intervention, raising the need for a new economic evaluation outside the trial context. The aim of this study was to evaluate the cost-effectiveness of the IRIS programme now that it has been implemented across the UK. Our estimates use up-to-date figures from an MRC phase IV observational pragmatic implementation study (12) on costs and effectiveness from routine clinical practice and the most up-to-date model input parameters, including a recently updated Cochrane review of domestic violence advocacy (13).

## Methods

*Overview of economic evaluation*

This was a cost–utility analysis, comparing IRIS with usual care in general practices. The outcome measure was quality-adjusted life years (QALYs), as recommended for economic evaluations in the UK (14). The main analysis was from a societal perspective, as many of the costs of DVA are borne outside the health system; we also estimated cost utility from an NHS perspective. Costs were calculated in 2015/16 UK£. We calculated costs and benefits over a 10-year time horizon, with future costs and outcomes discounted at an annual rate of 3.5% (14).

*Model structure*

We developed a Markov model (Figure1) based on the previous analysis (11). The model has five states and the cycle length was six months; this length was chosen as it reflects the average amount of time women stay in contact with DVA advocacy services. We have used a half-cycle correction (15) A hypothetical cohort of 10,000 women aged 16 years or older was


For peer review only - http://bmjopen.bmj.com/site/about/guidelines.xhtml

simulated moving between the states (Figure 1). Other than death, which is an absorbing state, women can transition between each of the other states 'Not abused', 'Abused but not identified', 'Abused and identified, seeing advocate educator', 'Abuse and identified, not seeing advocate educator'. As the hypothetical cohort of women aged 16 or older were considered eligible for the intervention, all results were reported as "per woman aged 16 or older registered to GP practice".

*Intervention*

The IRIS programme is a multi-component intervention that has been described in detail elsewhere (10, 11). In brief, it consists of two two-hour multidisciplinary training sessions, for the practice clinical team and one hour training for reception and ancillary staff. They are delivered jointly by an IRIS advocate educator from a local collaborating specialist DVA agency, alongside a clinician interested in DVA, the IRIS clinical lead. The advocate educator is central to the intervention, combining a training and support role to the practices with provision of advocacy to women referred. Other intervention components include a simple 4-question questionnaire, carried out by the healthcare practitioner, addressing different aspects of DVA (Humiliation, Afraid, Raped and Kicked), such as "within the last year, have you been afraid of your partner of ex-partner?", also known as the HARK template (16) in the electronic medical record triggered by entry of clinical problem codes (such as depression, anxiety, irritable bowel syndrome, pelvic pain and assault), an explicit referral pathway to a named IRIS advocate educator, and publicity materials about DVA visible in practices. Patients referred to the advocate educator are usually seen at the referring general practice, enhancing safety and confidentiality.

*Data collection and ethics approval*


For peer review only - http://bmjopen.bmj.com/site/about/guidelines.xhtml

Several different data sources were used in this study. Whenever possible, we have used observational data from the IRIS programme. These were collected by IRIS team members, liaising with advocacy agencies and local authorities. Given that we only use anonymized data, arising from the usual care of women, individual consent of women was not required. This research project was given exemption from NHS Research Ethics processes, as it was classified as service evaluation. When observational data were unavailable, we have chosen to use peer-reviewed published data that was relevant to general practice and the UK. Each relevant parameter and its source are described in detail below.

*Prevalence of domestic abuse*

The proportion of women aged 16 years or older experiencing abuse was estimated based on published epidemiological data. This was taken from a cross sectional study carried out by Richardson and colleagues in east London (17), which reported a prevalence of 0.17 or 17% in the population of women consulting a general practitioner or practice nurse. This is an estimate of the prevalence of DVA in general practice, generalizable for England.

*Transition probabilities*

There are eight transitions between states in the model. Transition probabilities were obtained using observational data from the IRIS programme, the MOSAIC (MOthers' Advocates In the Community) programme (10, 18), the Office for National Statistics (19, 20) and Health & Social Care Information Centre (21), and a Cochrane review (13), evaluating the reduction of any type of domestic abuse with any type of advocacy. Observational data were obtained from commissioned IRIS sites that have been running for two years or more, where there was at least one full-time equivalent advocate educator and 20 general practices trained. It included 6 clinical commissioning groups (CCGs) in northern England, south-west England



For peer review only - http://bmjopen.bmj.com/site/about/guidelines.xhtml

and London. Given the inclusion criteria, the sites represent the implementation of the programme. Table 1 provides the parameter values and their respective sources. Where no data were available, we have calculated estimates using the model calibration method described below.

*Model calibration*

Because of uncertainty surrounding transition probabilities from *Not abused* to *Abused but not identified* and *vice versa*, we used the prevalence of abuse (17%) estimated in Richardson and colleagues' study (17), to calibrate the model. The model was run for 3000 cycles, assuming that thereafter the number of women in each state would remain constant. This was based on our calculation of steady states. The transition probabilities from *Not abused* to *Abused but not identified* and *vice versa* were changed until the proportion of women in the *Not abused* state exactly reflected the observed prevalence (100-17=83%). The initial distribution of women in the three *Abused* states was also determined by this process.

*Utilities*

Each state in the Markov model was associated with a utility score, which consisted of a general measure of health-related quality-of-life (22), allowing us to measure QALYs associated with IRIS and the comparator based on the proportion of women in each health state in each of the 20 6-monthly cycles in the model, totalling 10 years. The utility score of women who were not abused was assumed to be 0.85 (23). Wittenberg and colleagues conducted a cross-sectional survey to estimate community preferences for health states resulting from intimate partner violence. Using a UK-based algorithm, they found the utility of women experiencing any abuse was 0.64. When the severity/frequency of violence was low, the mean utility was 0.65 and when the severity/frequency was moderate or severe the



For peer review only - http://bmjopen.bmj.com/site/about/guidelines.xhtml

mean utility was 0.63. For women who were abused in our model, we assumed this was moderate to severe, giving a utility score of 0.63 (24). For women seeing an advocate educator, we used the utility value of women with low abuse (0.65), implying that seeing an advocate educator slightly increased their quality-of-life scores. QALY gains were reported per woman aged 16 or older registered to GP practice.

*Costs*

We included: intervention costs, costs of onward referral, and costs associated with DVA (including costs to the UK National Health Service (NHS), lost economic output, costs to the criminal/civil justice system, and personal costs). Costs were also reported per woman aged 16 or older registered to GP practice.

One IRIS advocate educator typically provides training, support and advocacy services for 24 general practices at any one point in time. Intervention costs were calculated based on the actual budget of the IRIS programme in the six sites (including advocate educator salaries, travel, recruitment, laptop, telephone, publicity, clinician consultancy, evaluation and central management costs) at a total six month cost across all sites of £272,613. This was divided by the number of registered women aged 16+ in IRIS-trained general practices in these sites (n=595,902). Costs of onward referral from the advocate educator was based on the finding of contact time from the IRIS trial, in which an onward referral was given to 57% of women in contact with an advocate educator and 63% of these women accepted this referral. Therefore, although costs of onward referral were based on current budgets and salaries, the proportion of contact was obtained from the trial estimates. Total costs per onward referral were therefore £861. Taking into account the proportion of women given a referral and accepting it,



For peer review only - http://bmjopen.bmj.com/site/about/guidelines.xhtml

and inflating it to 2015/16 UK£, average costs of advocate educator contact per abused woman were £312.

Costs associated with intimate partner violence in the UK are described by Walby and Olive (8). In their report, costs of lost economic output, health services, criminal justice system, civil justice system, social welfare, personal costs, specialised services and physical/emotional impact were individually reported, and total costs were €13,732 million (£11 billion) in 2012. We excluded costs of physical/emotional impact (€6,614 million), as they were not financial costs, but consisted of monetary valuing of health status, which in cost-effectiveness models ought to be captured in terms of QALYs; these were also not included in the original cost-effectiveness analysis. The remaining costs were converted to UK£ and inflated to 2015/16. Total costs per six months were £2,933 million. Based on the 2015 Crime Survey for England and Wales, it was estimated that 1.3 million women experienced intimate partner violence in 2015/16 in the UK (3). Mean costs per abused woman were therefore £2,043. We assumed that the costs of intimate partner abuse are similar to the costs of abuse by other family members, and that the costs would not differ between identified or unidentified abuse. In sensitivity analyses we have allowed the costs of identified abuse to increase or decrease by 10% compared to abuse that was not identified; similarly the costs of *Abused and identified, seeing advocate educator* were allowed to increase or decrease by 25%.

*Cost-utility analysis*

Costs and utilities were applied to each health state. Total costs and QALYs for the hypothetical cohort were generated for the IRIS programme and the control group. The main outcome was the incremental costs per QALY gained. In the UK an intervention is generally


For peer review only - http://bmjopen.bmj.com/site/about/guidelines.xhtml

considered cost-effective when the incremental costs per QALY gained are less than £20,000 (14). We also presented the results of cost-effectiveness analysis in terms of incremental net monetary benefit (NMB). This was calculated as the mean incremental QALYs per woman registered at the general practice accruing to IRIS multiplied by the decision-makers' maximum willingness to pay for a QALY (assumed to be £20,000), minus the mean incremental cost per woman. Negative incremental NMBs indicate that usual care was preferred on cost-effectiveness grounds and positive incremental NMBs favour IRIS.

The cost-utility analysis was conducted using pooled national data, but we have also evaluated the cost-effectiveness at different local sites. We allowed all parameters, including costs and benefits, to vary across sites and reported them individually.

*Sensitivity analysis*

All parameters were varied in a one-way sensitivity analysis, using lower and upper limits based on 95% uncertainty intervals. We undertook a probabilistic sensitivity analysis, drawing random samples from the probability distributions of all parameters in 1,000 simulations. All uncertainty intervals were calculated based on the 2.5$^{th}$ and 97.5$^{th}$ percentiles of the distribution of all the 1000 values in the probabilistic sensitivity analysis. The interpretation of these is different to that of statistical analysis confidence intervals of clinical effects. In cost-effectiveness analysis, if an ICER has an uncertainty interval that crosses zero, it effectively means that the intervention can be cost-saving (negative value), cost-neutral (zero) or costly (positive value) per QALY gained. The proportion of simulations with an incremental cost per QALY gained below the cost-effectiveness threshold was calculated for different values, ranging from £0 to £50,000. The results were presented in a cost-effectiveness acceptability curve.



For peer review only - http://bmjopen.bmj.com/site/about/guidelines.xhtml

*Patient and Public Involvement (PPI)*

We did not directly include PPI in this study, but the data collected from local IRIS Programmes was developed with PPI.

## Results

*Base case*

Parameter values used in the base case analysis are shown in Table 1, which also includes the parameters used in the original trial to allow for a direct comparison. The main differences between the parameters for this study and the trial parameters lie in the transition probabilities relating to the health state of 'abuse but not identified' and its cost.

Over the ten-year time horizon, mean total costs per woman were £4,416 in the intervention group, compared to £4,430 in the control group (Table 2(a)). The IRIS programme therefore saves £14 per woman aged 16 and older registered to GP practices, from a societal perspective over 10 years. Total QALYs per woman were 0.001 higher in the intervention group (6.671) than in the control group (6.669). Because the intervention was associated with lower costs and greater effectiveness the incremental cost per QALY gained was negative (i.e. IRIS dominates current practice as it is both cost-saving and more effective than usual care) and the incremental NMB was positive (£42). The incremental NMB was also positive (£22) when using an NHS-only perspective (Table 2(b)).

Table 2 also presents the results for each site. The table shows that IRIS dominated current practice, from a societal perspective, in sites 1, 2, 3 and 4, with an incremental net monetary benefit (NMB) of £41, £89, £29 and £59 respectively. From a NHS perspective, only in site 1 did IRIS dominate current practice, although it was cost-effective, using the threshold advised by NICE of £20,000 per QALY gained, in sites 2 (ICER £2,585 per QALY gained), 3 (ICER


For peer review only - http://bmjopen.bmj.com/site/about/guidelines.xhtml

£3,055 per QALY gained) and 4 (ICER £8,317 per QALY gained). IRIS was found to be cost-effective (ICER £5,882 per QALY gained) and borderline cost-effective (ICER £21,229 per QALY gained) from a societal and NHS perspectives respectively in site 5, and it was not cost-effective from either perspective in site 6 (ICER £52,557 per QALY gained and ICER £64,427 per QALY gained respectively).

*Sensitivity analyses*

Across all sites combined, results were most sensitive to varying the transition probability from *Abused but not identified* to *Not abused*. When in the control arm this was varied from 0.049 to 0.051, the incremental NMB varied from £110 to -£26 (Figure 2). When it was varied similarly in the intervention arm, the incremental NMB varied from -£25 to £109. Figure 2 shows the 12 parameters that when varied had the highest impact on the incremental NMB.

Incremental costs and QALYs varied widely in probabilistic sensitivity analyses. The 95% uncertainty interval for incremental costs was -£151 to £37, for incremental QALYs it was -0.005 to 0.006 and for the incremental NMB it was -£247 to £351. Figure 3(a) shows a scatter plot of the incremental costs and incremental QALYs from the 1,000 simulations. The IRIS programme is cheaper and more effective than the absence of the programme (usual care), dominating current practice in 35% of the simulations and was dominated by the absence of the programme in 18% of the simulations. The IRIS programme was cost-effective in 61% of simulations when the cost-effectiveness threshold was £20,000 (Figure 3(b)).

## Discussion

*Summary*

For peer review only

We found that the IRIS GP training and service programme is likely to be cost-effective and cost-saving in the UK compared to usual care. The QALY gains associated with IRIS, which are average values for all eligible women aged 16 or over registered at a practice (and not, for example, those who have been abused), are small; these are balanced against an equally small incremental cost of the intervention. Interventions with small costs and small gains are not uncommon in public health: a well-known example is flu vaccination (25, 26).There is considerable uncertainty surrounding these results, but the probability that IRIS is cost-effective was more than 60% at the cost-effectiveness threshold commonly used in the UK. The cost-effectiveness acceptability curve is relatively flat, implying that the results from IRIS do not change much regardless of the threshold used. In our view the shape of the CEAC is entirely consistent with the 95% uncertainty intervals. The fact that these values are close to 50% reflects there is a high level of uncertainty, and the fact that the probability that IRIS is cost-effective is just higher than 50% reflects the fact that IRIS is (slightly) favoured over the alternative according to our base case estimates. IRIS was more cost-effective when costs were measured from a societal perspective as the cost savings from reducing DVA were higher. IRIS was also cost-effective when taking an NHS-only perspective. There was some variation in value for money between sites, which appears to be driven mainly by the different rates of identification and/or referral, although different local costs have also contributed.

*Comparison with existing literature*

We contacted researchers in the field and searched the NHS Economic Evaluations Database and the HTA Database at the Centre for Reviews and Dissemination (27) for cost-effectiveness analyses of DVA programmes using the search terms “domestic violence” and “cost*” (28/08/2017). We identified four economic impact studies, all using modelling methods: one based on the pilot of the IRIS trial (22), another based on the main trial (11), the



For peer review only - http://bmjopen.bmj.com/site/about/guidelines.xhtml

third based on an evaluation of independent domestic violence Advisors (IDVA) (28), and the fourth of a trial of cognitive trauma therapy for abused women who have left the abusive relationship (28). All the studies found the interventions cost-effective, despite uncertainty. Devine et al has reported a 75% probability of the DVA intervention being cost-effective (11), while Mallender et al reported 2 scenarios our of possible 5 in which the intervention is not cost-effective (28). Our findings are consistent with these previous studies. Our study is the only one that analyses the economic impact of a primary care-based programme implemented outside of trial settings.

*Strengths and limitations*

Our analysis has the strength of being based on a previously published cost-effectiveness model, updated with real-life data. Importantly, intervention costs and the probability of referral with IRIS were based on actual clinical practice, rather than in a research setting. We also had new data for the probability of identifying abuse and for what happened to women who were abused in current practice without the programme. However, it was not possible to update all parameter values. In particular, we were unable to update the utility value estimates, although in the sensitivity analysis, we have allowed these to vary and results were relatively stable. Costs of the intervention were calculated by dividing the total costs of the programme over all registered women in practices with the IRIS programme. Many of these women will never experience abuse and therefore cannot directly benefit from the programme. If programme costs were divided over women experiencing abuse only, mean costs per woman would be higher. However, the QALYs gained would also be higher, as these are also calculated for all women in the practices rather than just those who were abused. In fact we have attempted to calculate these results dividing cost and QALYs over women experiencing abuse and the final ICER was unchanged, as both the numerator and



For peer review only - http://bmjopen.bmj.com/site/about/guidelines.xhtml

denominator change by the same proportion. We did not include any impact of the IRIS programme on children exposed to DVA, as to our knowledge, there are no available cohort studies focusing on the cost and benefits of DVA interventions for this population which might mean that we have underestimated the programme's cost-effectiveness. This was also highlighted in the NICE economic analysis of interventions to reduce incidence and harm of DVA: "It can be expected there are likely to be additional benefits such as [to] the children and wider family members of victims of domestic violence (p.11) (28).

Another limitation is that we have used mainly data on short-term outcomes, although modelled long-term outcomes. There is unfortunately little data on long-term outcomes of DVA and the effect of advocacy, although it is generally agreed that effects last for a long time. This, however, bias our estimates against the intervention, implying our results are conservative.

*Implications for research and/or practice*

The IRIS programme is likely to be cost-effective and cost-saving when implemented in the real life of the in the UK National Health System. In order to decrease uncertainty around the cost-effectiveness estimates of IRIS and programmes like it, more data are needed on the utilities of women identified and women seeing an advocate and on long-term outcomes associated with DVA. Furthermore, future research should endeavour to understand the impacts and economic burden of DVA on exposed children, other family members and friends, as well as focus on collecting up-to-date utility values for women subject to DVA in each health state.

Finally, our study has shown that there is moderate variation in the value for money of IRIS across different sites, implying qualitative research could focus on identifying the causes of such variation, in order to reduce it.



For peer review only - http://bmjopen.bmj.com/site/about/guidelines.xhtml

For peer review only

**Authors' Contribution**

SM, CG, SE, AS and GF have designed the study. EB, TV, SM, FS and AD have developed the Markov model and carried out the analysis of data. AS, FES, SD, CR, NL and MJ have collected and validated the data. EB and SM have produced the initial draft. All authors have critically revised the manuscript and approved the final version.

**Competing Interests disclosure**

MJ has been paid by the IRIS project since 2007 for employment as an IRIS Advocate Educator and then as a National Implementation Manager. She is currently paid by IRISi, a social enterprise that is promoting the commissioning of the IRIS programme, for employment as Chief Executive. GF reports grants from National Institute for Health Research (NIHR), during the conduct of the study; and he is a non-executive board member of IRISi. All other authors disclose no competing interests.

**Funding and Acknowledgements**

This research was funded by the National Institute for Health Research (NIHR) Collaboration for Leadership in Applied Health Research and Care North Thames at Barts Health NHS Trust.

GF acknowledges the support of the National Institute for Health Research from a programme grant for applied research RP-PG-0614-20012 REPROVIDE (Reaching Everyone Programme of Research on Violence in diverse Domestic Environments).

The views expressed are those of the author(s) and not necessarily those of the NHS, the NIHR or the Department of Health.

For peer review only

We would like to thank our IRIS partners who deliver the programme in the sites, especially those in northern England, south-west England and London who took the time and effort to provide us with data.

**Data sharing**

The anonymised data used in this study can be obtained from the corresponding author.

For peer review only

## References

1. Assembly UG. Declaration on the Elimination of Violence against Women. UN General Assembly. 1993.

2. Garcia-Moreno C, Jansen HA, Ellsberg M, Heise L, Watts CH. Prevalence of intimate partner violence: findings from the WHO multi-country study on women's health and domestic violence. Lancet. 2006;368(9543):1260-9.

3. ONS. Intimate personal violence and partner abuse compendium. 2016.

4. Bonomi AE, Anderson ML, Reid RJ, Rivara FP, Carrell D, Thompson RS. Medical and psychosocial diagnoses in women with a history of intimate partner violence. Archives of internal medicine. 2009;169(18):1692-7.

5. Campbell JC. Health consequences of intimate partner violence. Lancet. 2002;359(9314):1331-6.

6. Tollestrup K, Sklar D, Frost FJ, Olson L, Weybright J, Sandvig J, et al. Health indicators and intimate partner violence among women who are members of a managed care organization. Prev Med. 1999;29(5):431-40.

7. Coid J, Petruckevitch A, Chung WS, Richardson J, Moorey S, Feder G. Abusive experiences and psychiatric morbidity in women primary care attenders. The British journal of psychiatry : the journal of mental science. 2003;183:332-9; discussion 40-1.

8. Walby S, Olive P. Estimating the costs of gender-based violence in the European Union. European Institute for Gender Equality; 2014.

9. European Innovation Partnership on A, Healthy Ageing APB, Mechanisms of the Development of Allergy WP, Global Alliance against Chronic Respiratory D, Bousquet J, Addis A, et al. Integrated care pathways for airway diseases (AIRWAYS-ICPs). The European respiratory journal. 2014;44(2):304-23.

10. Feder G, Davies RA, Baird K, Dunne D, Eldridge S, Griffiths C, et al. Identification and Referral to Improve Safety (IRIS) of women experiencing domestic violence with a primary care training and support programme: a cluster randomised controlled trial. Lancet. 2011;378(9805):1788-95.

11. Devine A, Spencer A, Eldridge S, Norman R, Feder G. Cost-effectiveness of Identification and Referral to Improve Safety (IRIS), a domestic violence training and support programme for primary care: a modelling study based on a randomised controlled trial. BMJ open. 2012;2(3).

12. Sohal A, Dowrick A, El-Shoghri F, Beresford L, Lewis N, Barbosa E, et al. Improving the healthcare response to domestic violence and abuse in primary care: protocol for

For peer review only

evaluation of a complex intervention's implementation into multiple general practices, including a phase IV observational segmented regression interrupted time series analysis BMJ Public Health. 2018 forthcoming.

13. Rivas C, Ramsay J, Sadowski L, Davidson LL, Dunne D, Eldridge S, et al. Advocacy interventions to reduce or eliminate violence and promote the physical and psychosocial well-being of women who experience intimate partner abuse. status and date: New search for studies and content updated (no change to conclusions), published in. 2015(12).

14. NICE NIoHaCE. Guide to the methods of technology appraisal 2013 [Available from: http://www.nice.org.uk/media/D45/1E/GuideToMethodsTechnologyAppraisal2013.pdf.

15. Naimark DM, Bott M, Krahn M. The half-cycle correction explained: two alternative pedagogical approaches. Medical Decision Making. 2008;28(5):706-12.

16. Sohal H, Eldridge S, Feder G. The sensitivity and specificity of four questions (HARK) to identify intimate partner violence: a diagnostic accuracy study in general practice. BMC family practice. 2007;8(1):49.

17. Richardson J, Coid J, Petruckevitch A, Chung WS, Moorey S, Feder G. Identifying domestic violence: cross sectional study in primary care. Bmj. 2002;324(7332):274.

18. Taft AJ, Small R, Hegarty KL, Watson LF, Gold L, Lumley JA. Mothers' AdvocateS In the Community (MOSAIC)--non-professional mentor support to reduce intimate partner violence and depression in mothers: a cluster randomised trial in primary care. BMC public health. 2011;11:178.

19. Statistics OoN. Crime Statistics, Focus on Violent Crime and Sexual Offences 2013/14 [Available from: http://www.ons.gov.uk/ons/rel/crime-stats/crime-statistics/focus-on-violent-crime-and-sexual-offences--2013-14/index.html.

20. Statistics OfN. Mortality Statistics: Deaths Registered in England and Wales 2013 [Available from: http://www.ons.gov.uk/ons/publications/re-reference-tables.html?edition=tcm%3A77-327590.

21. Centre HSCI. Numbers of Patients Registered at a GP practice January 2014

22. Horsman J, Furlong W, Feeny D, Torrance G. The Health Utilities Index (HUI®): concepts, measurement properties and applications. Health and quality of life outcomes. 2003;1(1):54.

23. Kind K, Hardman G, Macran S. UK Population norms for EQ-5D. University of York; 1999.

24. Wittenberg E, Lichter EL, Ganz ML, McCloskey LA. Community preferences for health states associated with intimate partner violence. Medical care. 2006;44(8):738-44.

For peer review only

25. Muennig PA, Khan K. Cost-effectiveness of vaccination versus treatment of influenza in healthy adolescents and adults. Clinical infectious diseases. 2001;33(11):1879-85.

26. Sander B, Gyldmark M, Aultman R, Aoki FY. Impact on health outcome and costs of influenza treatment with oseltamivir in elderly and high-risk patients. Journal of Medical Economics. 2004;7(1-4):67-83.

27. York Uo. Centre for Reviews and Dissemination [Available from: http://www.crd.york.ac.uk/CRDWeb/HomePage.asp.

28. Mallender J, Venkatachalam M, Onwude O, Jhita T. Economic analysis of interventions to reduce incidence and harm of domestic violence. London: National Institute for Health and Care Excellence. 2013.

For peer review only

**Table 1. Model input parameters: probabilities; utilities; and, costs.**

| **Parameter** | **Base case value** | **Lower limit** | **Upper limit** | **Distribution** | **Source** | **IRIS trial base value**[1] |
|---|---|---|---|---|---|---|
| ***Probabilities*** | | | | | | |
| Proportion of women experiencing abuse | 0.17 | 0.147 | 0.194 | Beta | (17) | 0.17 |
| *Starting distribution for women who are abused* | | | | | | |
| Abused and identified, seeing advocate educator | 0.003¶ | 0 | 0.0066 | Uniform | * | - |
| Abused and identified, not seeing advocate educator | 0.033¶ | 0 | 0.0660 | Uniform | * | - |
| Abused but not identified | 0.964¶ | - | - | Uniform | Complement | - |
| *Transition probabilities* | | | | | | |
| Not abused to Abused but not identified | 0.0037¶ | 0.0004 | 0.0106 | Dirichlet | * | 0.0075 |
| Not abused to Dead | 0.00551¶ | 0.0010 | 0.0136 | Dirichlet | (13, 15) | 0.0058 |
| Stay in Not abused | 0.9908¶ | - | - | Dirichlet | Complement | 0.9867 |
| Abused but not identified to Not abused (control) | 0.0500¶ | 0.0450 | 0.0553 | Dirichlet | * | 0.025 |
| Abused but not identified to Abused and identified, not seeing advocate educator (control) | 0.0027¶ | 0.0016 | 0.0040 | Dirichlet | IRIS-programme local sites | 0.0094 |



For peer review only - http://bmjopen.bmj.com/site/about/guidelines.xhtml

| | | | | | | |
|---|---|---|---|---|---|---|
| Abused but not identified to Abused and identified, seeing advocate educator (control) | 0.0005¶ | 0.0001 | 0.0011 | Dirichlet | IRIS-programme local sites | 0.0016 |
| Abused but not identified to Dead (control) | 0.00554¶ | 0.0039 | 0.0074 | Dirichlet | (13, 15) | 0.0059 |
| Stay in Abused but not identified (control) | 0.9444¶ | - | - | Dirichlet | Complement | 0.9581 |
| Abused but not identified to Not abused (intervention) | 0.0500¶ | 0.0450 | 0.0553 | Dirichlet | * | 0.025 |
| Abused but not identified to Abused and identified, not seeing advocate educator (intervention) | 0.0109¶ | 0.0086 | 0.0135 | Dirichlet | IRIS-programme local sites | 0.0207 |
| Abused but not identified to Abused and identified, seeing advocate educator (intervention) | 0.0056¶ | 0.0040 | 0.0076 | Dirichlet | IRIS-programme local sites | 0.0101 |
| Abused but not identified to Dead (intervention) | 0.00554¶ | 0.0039 | 0.0074 | Dirichlet | (6) | 0.0059 |
| Stay in Abused but not identified (intervention) | 0.9419 ¶ | - | - | Dirichlet | Complement | 0.9383 |
| Abused and identified, seeing advocate educator to Not abused | 0.1408¶ | 0.0707 | 0.2301 | Dirichlet | (18) | 0.0888 |
| Abused and identified, seeing advocate educator to Dead | 0.00554¶ | 0.0000 | 0.0309 | Dirichlet | (13, 15) | 0.0059 |
| Stay in Abused and identified, seeing advocate educator | 0.8536¶ | - | - | Dirichlet | Complement | 0.9053 |



For peer review only - http://bmjopen.bmj.com/site/about/guidelines.xhtml

| | | | | | | |
|---|---|---|---|---|---|---|
| Abused and identified, not seeing advocate educator to Not abused | 0.0781¶ | 0.0136 | 0.1912 | Dirichlet | (18) | 0.0717 |
| Abused and identified, not seeing advocate educator to Dead | 0.00554¶ | 0.0000 | 0.0438 | Dirichlet | (13, 15) | 0.0059 |
| Stay in Abused and identified, not seeing advocate educator | 0.9163¶ | - | - | Dirichlet | Complement | 0.9223 |
| ***Utilities*** | | | | | | |
| Not abused | 0.85 | 0.840 | 0.860 | Beta | (23) | - |
| Abused but not identified | 0.63 | 0.503 | 0.749 | Beta | (24) | - |
| Abused and identified, seeing advocate educator | 0.65 | 0.518 | 0.771 | Beta | (24) | - |
| Abused and identified, not seeing advocate educator | 0.63 | 0.503 | 0.749 | Beta | (24) | - |
| ***Costs*** | | | | | | |
| Costs of the intervention, per women registered, per 6 months | £0.46¶ | £0.01 | £1.69 | Gamma | IRIS-programme local sites | £0.55 |
| Cost of onward referral, once | £312¶ | £8 | £1127 | Gamma | IRIS-programme | £298 |



For peer review only - http://bmjopen.bmj.com/site/about/guidelines.xhtml

| | | | | | | |
|---|---|---|---|---|---|---|
| | | | | | local sites & (11) | |
| Cost of Abused but not identified | £2043 | £52 | £7536 | Gamma | (8) | £4721 |
| Weighted costs Abused and identified, seeing advocate educator | 1 | 0.75 | 1.25 | Gamma | Assumption | - |
| Weighted costs Abused and identified, not seeing advocate educator | 1 | 0.9 | 1.1 | Gamma | Assumption | - |

Costs are in 2015/16 UK£.
* Internal calculation based on model calibration.
¶ Value updated from Devine et al (11).
[1] Values obtained from Devine et al (11).



For peer review only - http://bmjopen.bmj.com/site/about/guidelines.xhtml

**Table 2. Base case results.**

| | (a) Societal perspective | | | (b) NHS-only perspective | | |
|---|---|---|---|---|---|---|
| **National IRIS (pooled results)** | **Costs** | **QALYs** | **Cost-effectiveness** | **Costs** | **QALYs** | **Cost-effectiveness** |
| Intervention (IRIS programme) | £4416 | 6.671 | | £1238 | 6. 671 | |
| Control (no programme) | £4430 | 6.669 | | £1232 | 6. 669 | |
| Difference (intervention vs. control) | -£14 | 0.001 | -ve (intervention dominates control) | £6 | 0.001 | £3913 per QALY gained |
| Incremental NMB* | | | £42 | | | £22 |
| **Local site 1** | | | | | | |
| Intervention (IRIS programme) | £4318 | 6.671 | | £1231 | 6.671 | |
| Control (no programme) | £4334 | 6.669 | | £1232 | 6.669 | |
| Difference (intervention vs. control) | -£16 | 0.001 | -ve (intervention dominates control) | -£1 | 0.001 | -ve (intervention dominates control) |
| Incremental NMB* | | | £41 | | | £26 |
| **Local site 2** | | | | | | |
| Intervention (IRIS programme) | £4305 | 6.673 | | £1240 | 6.673 | |
| Control (no programme) | £4333 | 6.670 | | £1232 | 6.670 | |
| Difference (intervention vs. control) | -£28 | 0.003 | -ve (intervention | £8 | 0.003 | £2585 per QALY |



For peer review only - http://bmjopen.bmj.com/site/about/guidelines.xhtml

| | | | | | | |
|---|---|---|---|---|---|---|
| | | | dominates control) | | | gained |
| Incremental NMB* | | | £89 | | | £54 |
| **Local site 3** | | | | | | |
| Intervention (IRIS programme) | £4325 | 6.671 | | £1235 | 6.671 | |
| Control (no programme) | £4334 | 6.670 | | £1232 | 6.670 | |
| Difference (intervention vs. control) | -£9 | 0.001 | -ve (intervention dominates control) | £3 | 0.001 | £3055 per QALY gained |
| Incremental NMB* | | | £29 | | | £17 |
| **Local site 4** | | | | | | |
| Intervention (IRIS programme) | £4326 | 6.672 | | £1253 | 6.672 | |
| Control (no programme) | £4334 | 6.669 | | £1232 | 6.669 | |
| Difference (intervention vs. control) | -£8 | 0.003 | -ve (intervention dominates control) | £21 | 0.003 | £8317 per QALY gained |
| Incremental NMB* | | | £59 | | | £30 |
| **Local site 5** | | | | | | |
| Intervention (IRIS programme) | £4337 | 6.670 | | £1244 | 6.670 | |
| Control (no programme) | £4332 | 6.669 | | £1232 | 6.669 | |
| Difference (intervention vs. control) | £4 | 0.001 | £5882 per QALY | £12 | 0.001 | £21229 per QALY |



For peer review only - http://bmjopen.bmj.com/site/about/guidelines.xhtml

| | | | | | | |
|---|---|---|---|---|---|---|
| | | | gained | | | gained |
| Incremental NMB* | | | £6 | | | £0 |
| **Local site 6** | | | | | | |
| Intervention (IRIS programme) | £4395 | 6.671 | | £1307 | 6.671 | |
| Control (no programme) | £4334 | 6.670 | | £1232 | 6.670 | |
| Difference (intervention vs. control) | £61 | 0.001 | £52557 per QALY gained | £75 | 0.001 | £64427 per QALY gained |
| Incremental NMB* | | | -£38 | | | -£52 |

NMB = net monetary benefit. QALY = quality-adjusted life year. Costs are in 2015/16 UK£. Numbers may not sum due to rounding.

*Measured at a willingness to pay for a QALY of £20 000.



For peer review only - http://bmjopen.bmj.com/site/about/guidelines.xhtml

**Figure 1. Health states and movement between health states in Markov model.**

Legend: The model starts with all women in either the 'Not abused' state or one of the states associated with abuse, based on the prevalence of DVA (see text). Women in the 'Not abused' state could stay in this state, move to 'Abused but not identified' or die from any cause. Once women were in the 'Abused but not unidentified' state, they could stay in that state, move back to 'Not abused', move to 'Abused and identified, seeing advocate' or 'Abused and identified, not seeing advocate' or die. Women in the 'Abused and identified' states could stay in these states, move back to 'Not abused' or die.



For peer review only - http://bmjopen.bmj.com/site/about/guidelines.xhtml

**Figure 2. Univariate sensitivity analysis.**

Legend: All analyses are as for the base case analysis with univariate adjustment of the parameters listed (see text). Results are point estimates of the incremental net monetary benefit (NMB) of the intervention vs. control. The incremental net monetary benefit is calculated at a maximum willingness to pay for a QALY of £20 000.

For peer review only



For peer review only - http://bmjopen.bmj.com/site/about/guidelines.xhtml

**Figure 3. Probabilistic sensitivity analysis.**

(a) **Scatter plot of incremental costs and incremental QALYs from 1000 simulations**
(b) **Cost-effectiveness acceptability curve showing the probability in percentage terms that the intervention is cost-effective vs. control at different values of the maximum willingness to pay for a QALY**

Legend: QALY = quality-adjusted life year. Costs are in 2015/16 UK£.

For peer review only



For peer review only - http://bmjopen.bmj.com/site/about/guidelines.xhtml

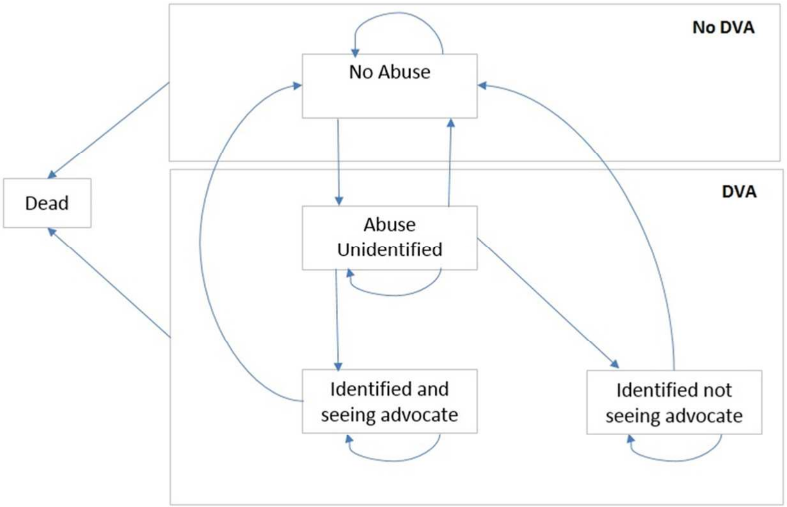


Figure 1. Health states and movement between health states in Markov model.
Legend: The model starts with all women in either the 'Not abused' state or one of the states associated with abuse, based on the prevalence of DVA (see text). Women in the 'Not abused' state could stay in this state, move to 'Abused but not identified' or die from any cause. Once women were in the 'Abused but not unidentified' state, they could stay in that state, move back to 'Not abused', move to 'Abused and identified, seeing advocate' or 'Abused and identified, not seeing advocate' or die. Women in the 'Abused and identified' states could stay in these states, move back to 'Not abused' or die.

160x90mm (300 x 300 DPI)

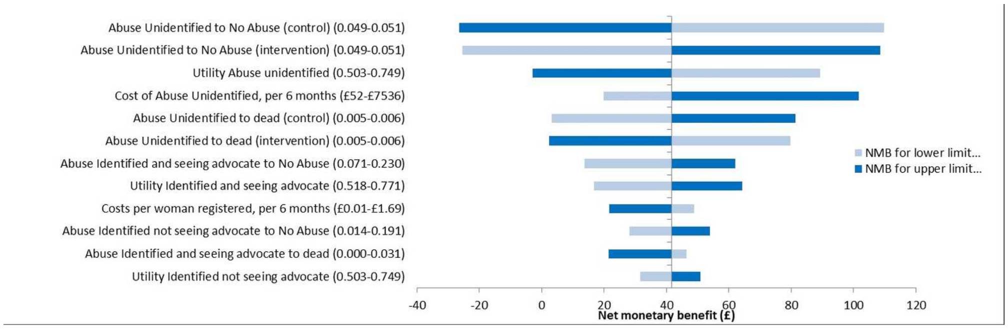


Figure 2. Univariate sensitivity analysis.
Legend: All analyses are as for the base case analysis with univariate adjustment of the parameters listed (see text). Results are point estimates of the incremental net monetary benefit (NMB) of the intervention vs. control. The incremental net monetary benefit is calculated at a maximum willingness to pay for a QALY of £20 000.

280x90mm (300 x 300 DPI)

er review only

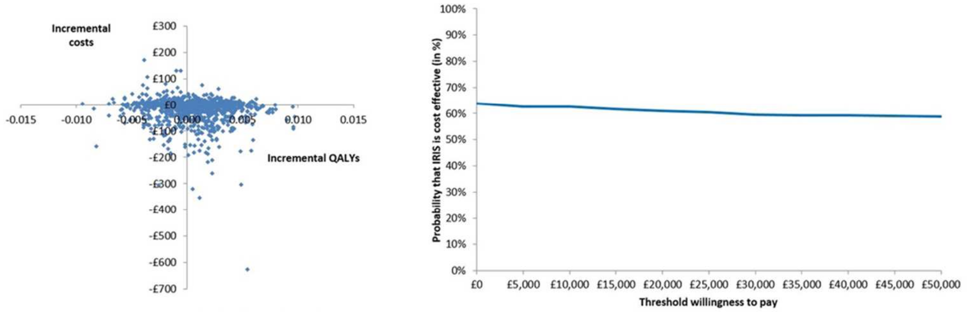


Figure 3. Probabilistic sensitivity analysis.
(a) Scatter plot of incremental costs and incremental QALYs from 1000 simulations
(b) Cost-effectiveness acceptability curve showing the probability in percentage terms that the intervention is cost-effective vs. control at different values of the maximum willingness to pay for a QALY
Legend: QALY = quality-adjusted life year. Costs are in 2015/16 UK£.

90x28mm (300 x 300 DPI)

For peer review only

## CHEERS checklist—Items to include when reporting economic evaluations of health interventions

| Section/item | Item No | Recommendation | Reported on page No/ line No |
|---|---|---|---|
| **Title and abstract** | | | |
| Title | 1 | Identify the study as an economic evaluation or use more specific terms such as "cost-effectiveness analysis", and describe the interventions compared. | page 1, line 1 to 4 |
| Abstract | 2 | Provide a structured summary of objectives, perspective, setting, methods (including study design and inputs), results (including base case and uncertainty analyses), and conclusions. | page 5, line 1 to page 6, line 12 |
| **Introduction** | | | |
| Background and objectives | 3 | Provide an explicit statement of the broader context for the study. | page 7, line 2 to 25 |
| | | Present the study question and its relevance for health policy or practice decisions. | page 7, line 25 to page 8, line 6 |
| **Methods** | | | |
| Target population and subgroups | 4 | Describe characteristics of the base case population and subgroups analysed, including why they were chosen. | page 8, line 22 to page 9, line 3; page 10, line 5 to 22 |
| Setting and location | 5 | State relevant aspects of the system(s) in which the decision(s) need(s) to be made. | page 10, line 17 to 20 |
| Study perspective | 6 | Describe the perspective of the study and relate this to the costs being evaluated. | page 8, line 12 to 14; page 29 to 31, table 2 |
| Comparators | 7 | Describe the interventions or strategies being compared and state why they were chosen. | page 8, line 10 |
| Time horizon | 8 | State the time horizon(s) over which costs and consequences are being evaluated and say why appropriate. | page 8, line 14 to 15 |
| Discount rate | 9 | Report the choice of discount rate(s) used for costs and outcomes and say why appropriate. | page 8, line 15 to 16 |
| Choice of health outcomes | 10 | Describe what outcomes were used as the measure(s) of benefit in the evaluation and their relevance for the type of analysis performed. | page 11, line 10 to 23 |
| Measurement of effectiveness | 11a | *Single study-based estimates:* Describe fully the design features of the single effectiveness study and why the single study was a sufficient source of clinical effectiveness data. | page 9, line 20 to page 10, line 3; page 10, line 5 to 22; page 11, line 1 to 8; pages 25 to 27, table 1 |
| | 11b | *Synthesis-based estimates*: Describe fully the methods used for identification of included studies and synthesis of clinical effectiveness data. | |
| Measurement and valuation of preference based outcomes | 12 | If applicable, describe the population and methods used to elicit preferences for outcomes. | not applicable |
| Estimating resources and costs | 13a | *Single study-based economic evaluation:* Describe approaches used to estimate resource use associated with the alternative interventions. Describe primary or secondary research methods for valuing each resource item in terms of its unit cost. Describe any adjustments made to approximate to opportunity costs. | |

For peer review only

| Section/item | Item No | Recommendation | Reported on page No/ line No |
|---|---|---|---|
| | 13b | *Model-based economic evaluation:* Describe approaches and data sources used to estimate resource use associated with model health states. Describe primary or secondary research methods for valuing each resource item in terms of its unit cost. Describe any adjustments made to approximate to opportunity costs. | page 9, line 20 to page 10, line 3; page 12, line 1 to page 13, line 11; pages 27 and 28, table 1 |
| Currency, price date, and conversion | 14 | Report the dates of the estimated resource quantities and unit costs. Describe methods for adjusting estimated unit costs to the year of reported costs if necessary. Describe methods for converting costs into a common currency base and the exchange rate. | page 28, table 1; page 8, line 14 |
| Choice of model | 15 | Describe and give reasons for the specific type of decision-analytical model used. Providing a figure to show model structure is strongly recommended. | page 8, line 18 to 25; figure 1 |
| Assumptions | 16 | Describe all structural or other assumptions underpinning the decision-analytical model. | page 11, line 14 to 23; page 10, line 25 to page 11, line 8; |
| Analytical methods | 17 | Describe all analytical methods supporting the evaluation. This could include methods for dealing with skewed, missing, or censored data; extrapolation methods; methods for pooling data; approaches to validate or make adjustments (such as half cycle corrections) to a model; and methods for handling population heterogeneity and uncertainty. | page 8, line 21 to 22; page 10, line 25 to page 11, line 8; |
| **Results** | | | |
| Study parameters | 18 | Report the values, ranges, references, and, if used, probability distributions for all parameters. Report reasons or sources for distributions used to represent uncertainty where appropriate. Providing a table to show the input values is strongly recommended. | page 14, line 17 to 20; page 25 to 28, table 1 |
| Incremental costs and outcomes | 19 | For each intervention, report mean values for the main categories of estimated costs and outcomes of interest, as well as mean differences between the comparator groups. If applicable, report incremental cost-effectiveness ratios. | page 14, line 21 to page 15, line 4; page 29 to 31, table 2; |
| Characterising uncertainty | 20a | *Single study-based economic evaluation:*Describe the effects of sampling uncertainty for the estimated incremental cost and incremental effectiveness parameters, together with the impact of methodological assumptions (such as discount rate, study perspective). | |
| | 20b | *Model-based economic evaluation:* Describe the effects on the results of uncertainty for all input parameters, and uncertainty related to the structure of the model and assumptions. | page 15, line 18 to page 16, line 6; figure 2; figure 3a and 3b |
| Characterising heterogeneity | 21 | If applicable, report differences in costs, outcomes, or cost-effectiveness that can be explained by variations between subgroups of patients with different baseline characteristics or other observed variability in effects that are not reducible by more information. | Page 15, line 6 to 15 |
| **6 Discussion** | | | |
| Study findings, limitations, | 22 | Summarise key study findings and describe how they support the conclusions reached. Discuss limitations | page 16, line 9 to page 19, line 5 |

For peer review only

| Section/item | Item No | Recommendation | Reported on page No/ line No |
|---|---|---|---|
| generalisability, and current knowledge | | and the generalisability of the findings and how the findings fit with current knowledge. | |
| **Other** | | | |
| Source of funding | 23 | Describe how the study was funded and the role of the funder in the identification, design, conduct, and reporting of the analysis. Describe other non-monetary sources of support. | page 20, line 14 to 20 |
| Conflicts of interest | 24 | Describe any potential for conflict of interest of study contributors in accordance with journal policy. In the absence of a journal policy, we recommend authors comply with International Committee of Medical Journal Editors recommendations. | page 20, line 7 a 12 |

For consistency, the CHEERS statement checklist format is based on the format of the CONSORT statement checklist

For peer review only
